# ZSTK474 targeting PIK3R3 inhibits the Wilms’ tumor through G0 / G1 phase arrest

**DOI:** 10.1371/journal.pone.0312178

**Published:** 2024-10-28

**Authors:** Maoxian Li, Jiayan Liu, Liming Jin, Tao Mi, Zhaoxia Zhang, Chenghao Zhanghuang, Mujie Li, Jinkui Wang, Xin Wu, Zhaoying Wang, Zhang Wang, Dawei He

**Affiliations:** 1 Department of Urology, National Clinical Research Center for Child Health and Disorders, Ministry of Education Key Laboratory of Child Development and Disorders, Chongqing Key Laboratory of Pediatrics, International Science and Technology Cooperation Base of Child Development and Critical Disorders, Children’s Hospital of Chongqing Medical University, Chongqing, P.R China; 2 Department of Pediatric Surgery, Chengdu Women’s and Children’s Central Hospital, School of Medicine, University of Electronic Science and Technology of China, Chengdu, Sichuan Province, China; The University of Tennessee Health Science, UNITED STATES OF AMERICA

## Abstract

**Purpose:**

Wilms’ tumor (WT), also known as nephroblastoma, is the predominant form of primary malignant renal cancer. The unfavorable prognoses linked to anaplastic nephroblastoma and recurrent nephroblastoma emphasize the crucial requirement for the exploration of innovative treatment modalities for WT.

**Methods:**

Our study conducted one-way Cox regression and Kaplan-Meier analyses using TARGET-WT nephroblastoma data to identify differentially expressed genes in nephroblastoma and evaluate their prognostic relevance. Utilizing the Connectivity Map database, ZSTK474 emerged as a viable therapeutic option for WT. The effect of ZSTK474 on WT and related underlying mechanisms were further investigated through in vitro and in vivo investigations.

**Results:**

The in vivo experiment results indicated that ZSTK474 effectively inhibited subcutaneous tumor growth in WT mice. CCK-8 assays revealed two nephroblastoma cell lines exhibited half-inhibitory concentrations of 2μM and 2.51μM for ZSTK474, respectively. ZSTK474 was shown to inhibit the migration and invasion capabilities of WT cells in both Transwell and wound healing assays. Flow cytometry apoptosis and TUNEL assays demonstrated that ZSTK474 induced apoptosis in WT cells. Cell cycle analysis revealed that ZSTK474 led to the induction of G0/G1 phase arrest. Sequencing of ZSTK474-treated WiT49 cells suggested that the impact of ZSTK474 on WT might be mediated by the PI3K/Akt pathway, specifically by inhibiting PIK3R3. Knock-down of PIK3R3 confirmed that ZSTK474 downregulated PIK3R3, reducing Akt phosphorylation, cyclin D and CDK4 levels and elevating P21 expression in nephroblastoma cells. However, current research has limitations, including a lack of understanding of the long-term effects and potential resistance mechanisms of new therapies.

**Conclusion:**

This research provides insight into the potential of ZSTK474 and other PI3K inhibitors for treating nephroblastoma.

## 1. Introduction

Wilms tumors (WT), also known as nephroblastoma, arise from embryonic kidney remnants and are the most prevalent primary malignant neoplasm impacting pediatric kidneys, accounting for 6% of all childhood cancers [1, 2]. Recent advancements in medical technology have significantly improved the long-term survival rates of WT [3–5]. However, aggressive WT subtypes, characterized by germ type, anaplastic histology, or 1q gain, pose ongoing therapeutic challenges [4, 6–10]. The 5-year survival rates for anaplastic and recurrent WT remain notably low, particularly in recurrent and high-grade anaplastic histology cases, where survival rates drop below 50% [11–15]. A study on nephroblastoma survivors treated over 25 years revealed that 65% of patients experienced chronic health issues, with 24% facing severe conditions [1]. Patients undergoing radiation therapy are at an increased risk of developing secondary malignancies and reduced fertility [6, 16–18].

Despite notable progress in multimodal therapies for WT, the development of novel chemotherapy agents for pediatric malignancies encounters substantial hurdles, such as low approval rates for clinical studies, time-consuming procedures, and elevated expenses [19]. Targeted therapies and immunotherapies have emerged as promising approaches for precise and less invasive treatment of nephroblastoma. Improving chemotherapeutic agents to target tumor cells and selectively reduce toxic effects is vital for enhancing pediatric nephroblastoma patients’ quality of life and survival rates [20–22]. Thus, conducting thorough research on personalized treatments for nephroblastoma and innovating chemotherapeutic agents are imperative endeavors.

The TARGET database, initiated by the National Cancer Institute (NCI), serves as an online repository to facilitate the development of targeted cancer therapeutics. Through sequencing and microarray technologies, the database enables the characterization of childhood cancers’ genomes, transcriptomes, and epigenetics, generating specific molecular alteration profiles. By examining perturbation-driven gene expression data stored in the Connectivity Map (CMap) database, researchers can uncover significant insights into biological mechanisms [22]. Researchers identify potential therapeutic agents by examining differentially expressed genes (DEGs) in healthy and diseased tissues and comparing them with the expression patterns of various substances obtained from the CMap database. This analysis results in a collection of substances determined by connectivity scores ranging from -1 to 1 [23]. Compounds showing a negative correlation with the DEGs associated with a specific disease are given priority for further investigation in therapeutic interventions [23–25]. The utilization of gene expression profiling and similarity analyses within the CMap database elucidates relationships among diseases, genes, and drugs, offering valuable insights for the development of innovative medical treatments.

ZSTK474 is a pan-class I PI3K inhibitor that effectively targets all four catalytic isoforms of class I PI3K with high specificity compared to other PI3K classes and protein kinases [26]. The inhibition of the PI3K/Akt signaling by ZSTK474 is attributed to its interactions with PI3K targets, inducing cell cycle arrest, anti-angiogenic effects, and anti-proliferative actions in various preclinical models [27–29]. Moreover, it has been demonstrated that ZSTK474 has efficacy against various adult tumor types, including lung cancer [26], colorectal cancer, prostate cancer [30, 31], breast cancer [32], leukemia [33], and glioblastoma [34]. Hence, ZSTK474 displays promise as an anti-cancer agent [26]. Despite this, there is insufficient research on implementing ZSTK474 in pediatric solid tumors.

This study employed the TARGET database to examine gene expression patterns linked to nephroblastoma and to identify DEGs associated with WT prognosis. By cross-referencing these patterns with the references of the CMap database, the results indicated that ZSTK474 exhibited the highest negative correlation coefficient with the disease among the tested compounds. The prediction indicates that ZSTK474 emerged as a promising therapeutic candidate for WT. Then, in vitro and in vivo experiments were conducted to examine the role of ZSTK474 in WT. Additionally, a thorough investigation was undertaken using cellular transcriptome sequencing technology to uncover the mechanism underlying the anti-WT effect of ZSTK474, presenting innovative insights and potential therapeutic approaches for nephroblastoma.

## 2. Materials and methods

### 2.1. Bioinformatics

#### 2.1.1. Nephroblastoma dataset

Data in the RNA-Seq-HTSeq-Counts format and relevant clinical information such as demographic data, tumor stage, and prognosis were acquired from the TARGET database for the nephroblastoma (TARGET-WT) projects. The date were accessed at 05/26/2023. The study included 130 samples, including 124 tumor and 6 non-tumor control samples.

#### 2.1.2. Screens for differential and prognostic genes

Differential gene expression and identification of prognostic genes were performed by comparing normal and tumor samples utilizing R software (edgeR package). Gene expression data in count format were used for differential analysis. Genes that satisfied the conditions of P<0.05 and |Log2FC|>1 were considered statistically significant. False Discovery Rate (FDR) correction was applied to adjust the p-values. Visualization was accomplished by generating heat maps and volcano plots with ggplot2. For survival analysis, gene expression data were represented as Transcripts Per Million (TPM). A one-way Cox regression analysis was performed for all genes with p-values < 0.05, utilizing R software with the SURVIVAL package. In the Cox risk model analysis, the hazard ratio (HR) is crucial. An HR over 1 with a p-value under 0.05 signals a risk factor, whereas an HR under 1 indicates a protective factor. This interpretation is included in the results. Genes were stratified into high and low expression groups according to median expression values, and Kaplan-Meier curves were subsequently generated.

#### 2.1.3. Drug prediction

By conducting genome-wide transcription profiling, the CMap database provides an in-depth representation of diverse biological states, encompassing diseases, physiological processes, drug responses, and perturbations. Applying GSEA algorithms elucidates associations between drug-related pathways and treatable conditions, revealing potential synergistic or antagonistic effects. This investigation integrates overexpressed genes associated with adverse outcomes in nephroblastoma into the CMap database to predict prospective therapeutic strategies.

#### 2.1.4. GO-KEGG and GSEA enrichment analyses

The clusterProfile package in R facilitated the analysis of differential gene expression by incorporating the GO and KEGG databases (P<0.05). Moreover, GSEA analysis was performed utilizing the MSigDB Collection gene set database, with genes ranked based on Hazard Ratio (HR) values.

### 2.2. Clinical specimens

The study was approved by the Institutional Review Board (IRB) of Children’s Hospital of Chongqing Medical University (approval number: IRB #2022–50). Prior to enrollment, patients and their guardians provided informed consent. Between June 2018 and June 2021, Clinical specimens were collected from pediatric patients with nephroblastoma at the Children’s Hospital of Chongqing Medical University. Following the initial tumor resection, two pathologists from the Department of Pathology conducted histological confirmation of the nephroblastoma diagnosis. Eight pairs of tumor and adjacent normal tissue samples from nephroblastoma patients were subjected to high-throughput RNA sequencing. The RNA-seq data (accession number GSE197047) was deposited in the GEO database.

### 2.3. Animal experiments and pathological histology

#### 2.3.1. In vivo experiments

The in vivo experiments were approved by the Institutional Animal Care and Use Committee of Chongqing Medical University (approval number: CHCMU-IACUC20220323001). After being sourced from the Animal Research Center at Chongqing Medical University, BALB/C nude mice (male, 3 weeks old) were housed in stainless steel cages in a ventilated animal room. The room temperature in the housing facility was maintained at 24°C, with a relative humidity of 60% and a 12-h light/dark cycle. Distilled water and sterilized food were available ad libitum. The mice were acclimated for a week. Injection of 2×10^6^ WiT49 cells, suspended in 100μL of DMEM, into the left axilla of the mice was used to induce subcutaneous nephroblastoma xenograft tumors. Once the tumor volume reached 90–100 mm^3^, the mice were segregated into four groups (n = 6 in each group). Except for one control group that was given PBS, three groups were given daily doses of ZSTK474 (MCE, China) at 25, 50, and 100 mg/kg over a two-week period. Tumor size and body weight of tumor-bearing mice were monitored throughout the study, and no mice died before meeting criteria for euthanasia. The mice were anaesthetised using 3% isoflurane, blood was taken from the eye socket and then executed by decapitation. Standard blood tests and serum analyses were performed to evaluate toxicity, focusing on liver, cardiac, and renal markers for tumor-bearing mice of each group. Tumor weights were recorded, and samples of various organs and tumors were collected for further analysis.

#### 2.3.2. Hematoxylin and eosin (H&E) stain

Fresh tumor samples were fixed in paraformaldehyde for 72 hours. The samples were processed through paraffin embedding and subsequently sectioned at a thickness of 4 μm. The subsequent procedures comprised hydration with xylene and alcohol, staining with hematoxylin and eosin, and dehydration through immersion in graded alcohol and xylene. Subsequently, images were acquired using light microscopy from Nikon, Japan.

#### 2.3.3. Immunofluorescence stain

Following fixation in 4% paraformaldehyde for more than 24 hours, tumor tissues underwent dehydration and paraffin embedding and were then sectioned into 4-μm slices. The sections were then subjected to thermal antigen retrieval by treating them with sodium citrate buffer and subsequently exposing them to microwave radiation. Following dewaxing, the sections were blocked using 0.5% bovine serum albumin (BSA) and left to incubate with primary antibodies overnight at 4°C. Primary antibodies used for immunofluorescence (diluted in 0.5% BSA at a 1:200 ratio) were PCNA (ZENBIO, China), VEGF (ZENBIO, China), MMP9 (Affinity Biosciences, China), and MMP2 (Affinity Biosciences, China). Following washing, sections were exposed to a fluorescent Cy3-conjugated secondary antibody, stained with Hoechst, and visualized using a Nikon C2 laser confocal microscope.

### 2.4. Cell lines and cell culture

WiT49 and HEK-293T cells were obtained from the Cell Bank (Chinese Academy of Sciences, Shanghai, China). WT-CLS1 cells were provided by the Capital Institute of Pediatrics (Beijing, China). WiT49 exhibited anaplastic characteristics, whereas WT-CLS1 demonstrated metastatic properties [35]. The cells were cultured in DMEM media (MeilunBio, MA0212, China) supplemented with 10% fetal bovine serum (Corning, 35-076-CV, USA), penicillin, and streptomycin, in a 5% CO2 environment at 37°C. Once the cells have attached to the surface, continue by changing the culture medium every two days. When the cells are 80% to 90% fused, either passage them or freeze them for storage.

### 2.5. Cell proliferative activity

The assessment of cell proliferation was conducted utilizing the CCK-8 assay sourced from MCE (HY-K0301, USA). Following detachment utilizing 0.25% trypsin, WiT49 (2.5×10^3^) and WT-CLS1 (3×10^3^) cells were cultured in 96-well plates. Following adhesion, cells were subjected to varying doses of ZSTK474 (0–32 μM) for a duration of 48 hours. Following a 2-hour incubation with culture medium (90 μL) and CCK-8 reagent (10 μL) at 37°C, the measurement of the absorbance at 450 nm was conducted to measure the IC50 value of ZSTK474. Following this, the cells were treated with ZSTK474 at concentrations equivalent to 1/2, 1, and 2 times the IC50 value. Growth curves were subsequently plotted at 0, 24, 48, and 72 hours post-incubation.

### 2.6. Cell migration ability assay

WT cells (2×10^5^ cells per well) were cultured in 6-well plates in a 5% CO2 environment at 37°C until confluence was achieved. A consistent wound was generated on the cellular surface, and any detached cells were eliminated using PBS. After this, WiT49 and WT-CLS1 cells were subjected to varying concentrations of ZSTK474 in a serum-free DEME medium. After a 48-hour incubation, cell migration was assessed at 0, 24, and 48 hours under a Nikon microscope. The scratch width measurement was performed using ImageJ.

### 2.7. Cell invasiveness assay

The assessment of cell invasiveness was performed through a Transwell assay on plates coated with matrix gel. When seeded on plates, WiT49 and WT-CLS1 cells were positioned in the lower chamber with a 10% FBS medium, while different concentrations of ZSTK474 in a serum-free medium were placed in the upper chamber. Cells were fixed and stained with crystal violet after 24 and 48 hours of treatment. Cell invasiveness was quantified using ImageJ software while observing under a light microscope.

### 2.8. Apoptosis by flow cytometry and TUNEL assay

The assessment of apoptosis was carried out using the Annexin V Apoptosis Kit (BDPharmingen, 5565547, USA). WiT49 and WT-CLS1 cells were cultured in 6-well plates for 12 hours. Subsequently, they were exposed to different concentrations of ZSTK474 for 48 hours and then analyzed using flow cytometry. FlowJo 10.8.1 software was utilized for data analysis.

In WT cells, apoptosis-induced DNA damage was identified through TUNEL staining in the low, medium, and high-dose intervention groups. After a 12-hour incubation in 24-well plates, cells were treated with varying concentrations of ZSTK474 for 48 hours. The cells were then subjected to TUNEL staining according to the manufacturer’s instructions. The cell nuclei were counterstained with DAPI and then mounted utilizing an anti-fade mounting medium. The Nikon C2 microscope was employed for image acquisition.

### 2.9. Cell cycle assay

The assessment of cell cycle progression after exposure to different concentrations of ZSTK474 was conducted using the BD Cell Cycle Assay Kit from Dojindo, Japan. In accordance with standardized procedures, WT-CLS1 and WiT49 cells were cultured for 48 hours, then fixed in 75% ethanol for an additional 48 hours. Following this, the cells were centrifuged, resuspended twice in PBS, and stained with propidium iodide and RNase at 37°C for 30 minutes. Lastly, cell cycle distribution was assessed through flow cytometry.

### 2.10. RNA sequencing and bioinformatics analysis

Sequencing was performed on the transcriptomes of WiT49 cells following treatment with ZSTK474 at concentrations of 0 μM or 2 μM, with three replicates for each treatment. Differential analysis was conducted utilizing R (edgeR package). Significance was determined as p < 0.05 and |Log2FC| > 1. Heat maps and volcano plots were plotted to visualize DEGs. Moreover, the clusterProfiler package in R was utilized for conducting GO functional enrichment and KEGG pathway analysis of DEGs (P < 0.05).

### 2.11. Reverse transcription polymerase chain reaction (RT-PCR)

Total RNA was extracted from WiT49 and WT-CLS1 cells utilizing TRIzol reagent (Invitrogen, CA, USA) and subsequently subjected to RT-PCR following the protocols of RTMasterMix (MCE, NJ, USA). Utilizing the SYBR Green RT-PCR Master Mix from MCE in NJ, USA, mRNA quantification was conducted on the Bio-Rad CFX ConnectTM Real-Time System. The relative expressions of NRG4, CDKN1A, FGF18, PIK3R3, and PDGFRB were measured using GAPDH as the internal reference gene. The primer information is summarized in Table 1.

**Table 1:** The sequence of primers used in this study.

Gene name	forward(5’-3’)	reverse(5’-3’)
PIK3R3	CCTGAATGTCTGGCTGGGAATTA	AGAGCAAGCATAGCATCCTTTCT
CDKN1A	CTGTCACTGTCTTGTACCCTTGT	CCCAGCAGAGGAACCACTACTA
PDGFRB	CCAATGAGGGTGACAACGACTAT	ATGGTTGAGGAGGTGTTGACTTC
FGF18	GACAAGTATGCCCAGCTCCTAGT	TTCTCGATGAACACACACTCCTT
NRG4	TGGCGGTCCTAGTAACACTTATC	GCTCGTCTCTACCAGGTTGATAT
GAPDH	TGTCACACGCTTTTGGGGTTT	CCTGGAAGATGGTGATGGGATT

### 2.12. RNA interfering

Tsingke Biotechnology Co., Ltd. (China) provided NC siRNA and PIK3R3-targeting siRNA (Table 2). WiT49 (3×10^5^) and WT-CLS1 (3×10^5^) cells were cultured in 6-well plates and incubated in Opti-MEM medium for 30 minutes before transfection. The oligomers were introduced into the cells using Lipofectamine 2000 reagent per the manufacturer’s guidelines. Following an 8-hour incubation, the medium was switched to a complete medium. Prior to harvesting, the cells were allowed to grow for an additional 48 hours.

**Table 2:** siRNA sequence.

Target gene name	sense(5’-3’)	antisense(5’-3’)
PIK3R3	CCGAGAUCAACACCUUGUA(dT)(dT)	UACAAGGUGUUGAUCUCGG(dT)(dT)

### 2.13. Western blot

The protein lysis solution containing 1% PMSF was used to lyse both WiT49 and WT-CLS1 cells treated with ZSTK474. After centrifugation at 12000g for 20 minutes, the protein content was quantified using a BCA assay kit (Thermo, China). Subsequently, 10 μg of protein underwent electrophoresis on a 6%-12.5% SDS-PAGE gel (EpiZyme, China) and was then transferred to a PVDF membrane (Millipore, ISEQ00010, USA) via semi-dry electrotransfer. After three TBST washes, the PVDF membranes were exposed to primary antibodies (Akt, MMP2, MMP9, PI3K, p-PI3K, p-Akt, PCNA, VEGF, P21, CyclinD, CDK4, GAPDH from ZENBIO, Chengdu, China; PIK3R3 from Proteintech, Wuhan, China) overnight at a 1:1000 dilution and 4°C. After being washed three times in TBST, the PVDF membrane was incubated with secondary antibodies (1:20000, ZENBIO, China) for one hour at room temperature. The Bio-RAD analyzer and Image Lab software were utilized for imaging and analysis.

### 2.14. Statistics

Animal experiments were conducted once, while phenotypic experiments were repeated at least three times for each experiment. All data were tested for normality prior to analysis, and data that followed a normal distribution were presented as mean ± standard deviation. Statistical analysis of the data was performed using GraphPad Prism version 9.0. Gene expression differences in paired tissue samples were analyzed using a paired t-test for normally distributed data and a Wilcoxon paired test for non-normally distributed data. Comparisons between two groups were conducted using an independent samples t-test, while comparisons among multiple groups were performed using one-way ANOVA. A p-value of less than 0.05 was considered statistically significant.

## 3. Results

### 3.1. Utilizing prognostic-related genes for bioinformatics analysis and drug prediction

**Fig 1:**
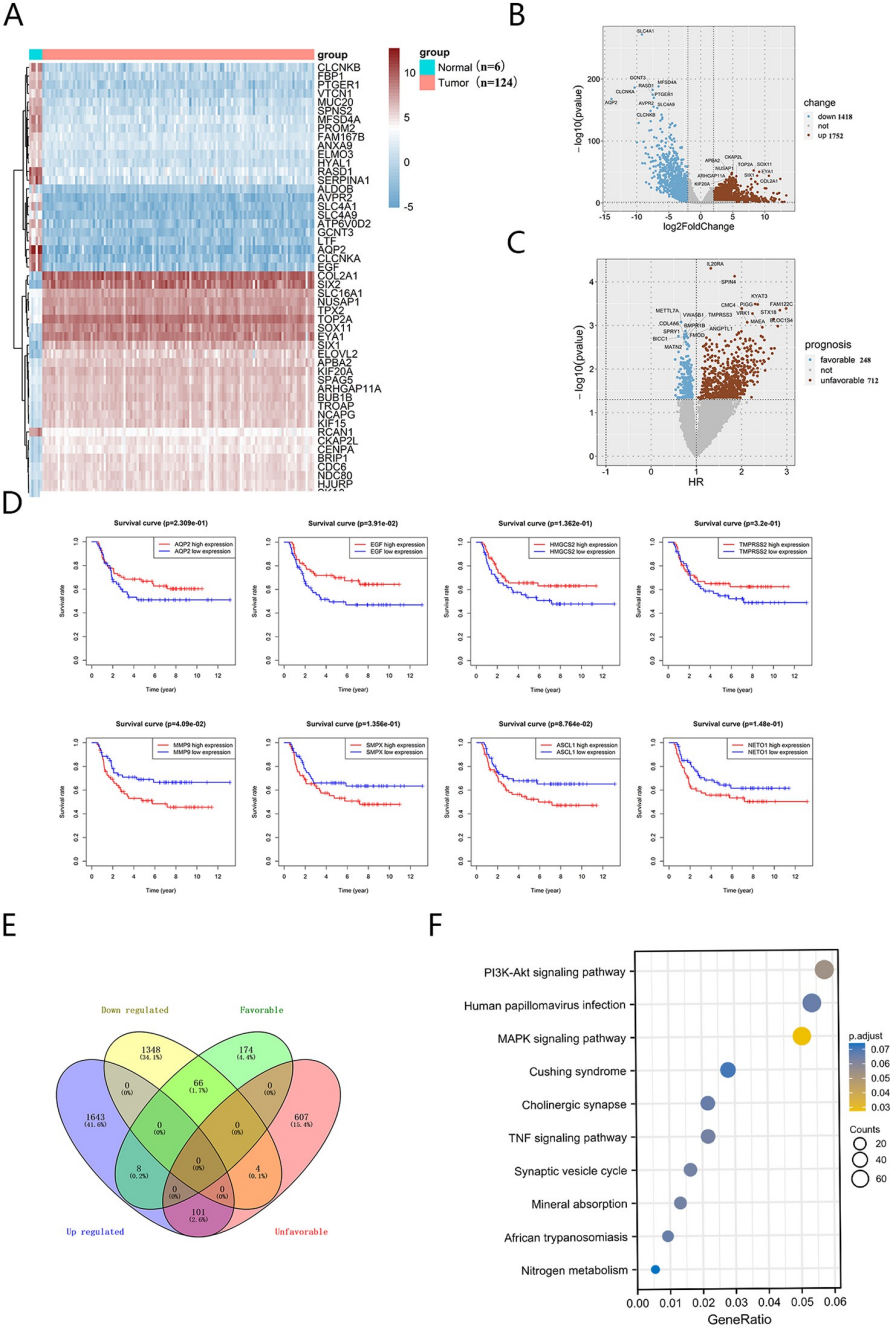
Bioinformatics analysis of WT prognosis-related genes and drug prediction. (A) Heatmap of differential genes from 124 tumors and 6 paratumors. (B-C) Volcano map of differential and prognosis-related genes in WT. (D) KM visualization of prognosis-related genes. (E) Venn diagram of differential genes versus and prognosis-related genes. (F) KEGG analysis of differential genes.

Gene expression analysis in nephroblastoma revealed 3170 DEGs, comprising 1418 down-regulated and 1752 upregulated genes (Fig 1A and 1B). A total of 960 genes were found to be significantly associated with prognosis (p < 0.01) through one-way Cox regression analysis, including 248 with a favorable prognosis (HR < 1) and 712 with an unfavorable prognosis (HR > 1) (Fig 1C). Prognosis-related genes were visualized using Kaplan-Meier analysis (Fig 1D) and overlapped with the DEGs. A subset of 101 upregulated genes, correlated with poor prognosis, was subjected to analysis using the CMap database (Fig 1E). The results indicated that ZSTK474 exhibited the highest negative correlation coefficient with the disease, leading to the discovery of ZSTK474, a promising PI3K inhibitor for nephroblastoma treatment. Additionally, KEGG analysis established a link between DEGs in nephroblastoma and the PI3K/Akt pathway (Fig 1F).

### 3.2. Aberrant activation of the PI3K/Akt pathway in WT

**Fig 2:**
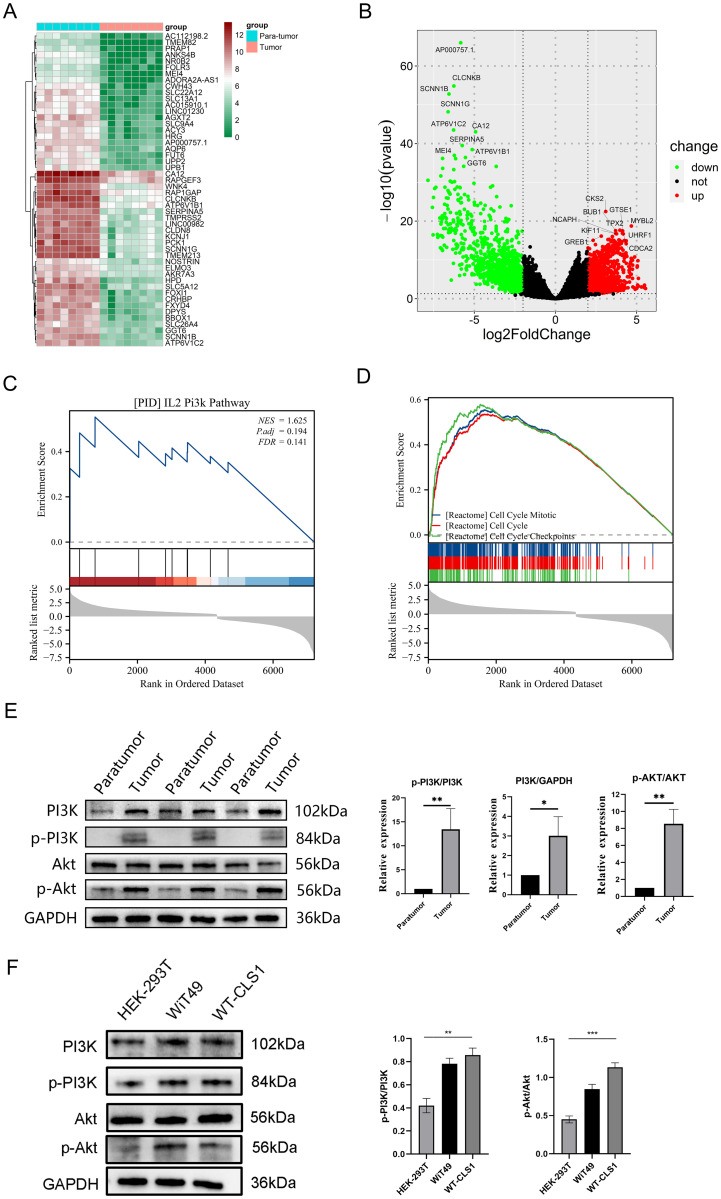
PI3K/Akt signaling pathway is abnormally activated in WT. (A) Heatmap of differential genes. (B) Volcano map of differential genes. (C and D) GSEA analysis of differential genes. (E) PI3K, p-Pi3k, p-Akt, and Akt expression in clinical samples from WT (n = 3). (F) PI3K, p-Pi3k, p-Akt, and Akt expression in WT cells and HEK-293T. ** P < 0.01, * P < 0.05.

Transcriptome sequencing was performed on eight human nephroblastoma samples to characterize gene expression profiles in DEGs, comprising 828 upregulated and 1114 down-regulated genes (Fig 2A and 2B). GSEA revealed significant enrichment in the cell cycle and PI3K/AKT pathways (Fig 2C and 2D). Further investigation included the evaluation of PI3K, Akt, phosphorylated PI3K, and phosphorylated Akt expression levels in WT tissue samples and cell lines. Western blot analysis demonstrated elevated levels of p-PI3K/PI3K and p-Akt/Akt in WT tumor tissues (Fig 2E). Similarly, WiT 49 and WT-CLS1 cells exhibited elevated levels of p-Akt/Akt and p-PI3K/PI3K relative to HEK-293T cells (Fig 2F).

### 3.3. Assessment of the safety of ZSTK474

**Fig 3:**
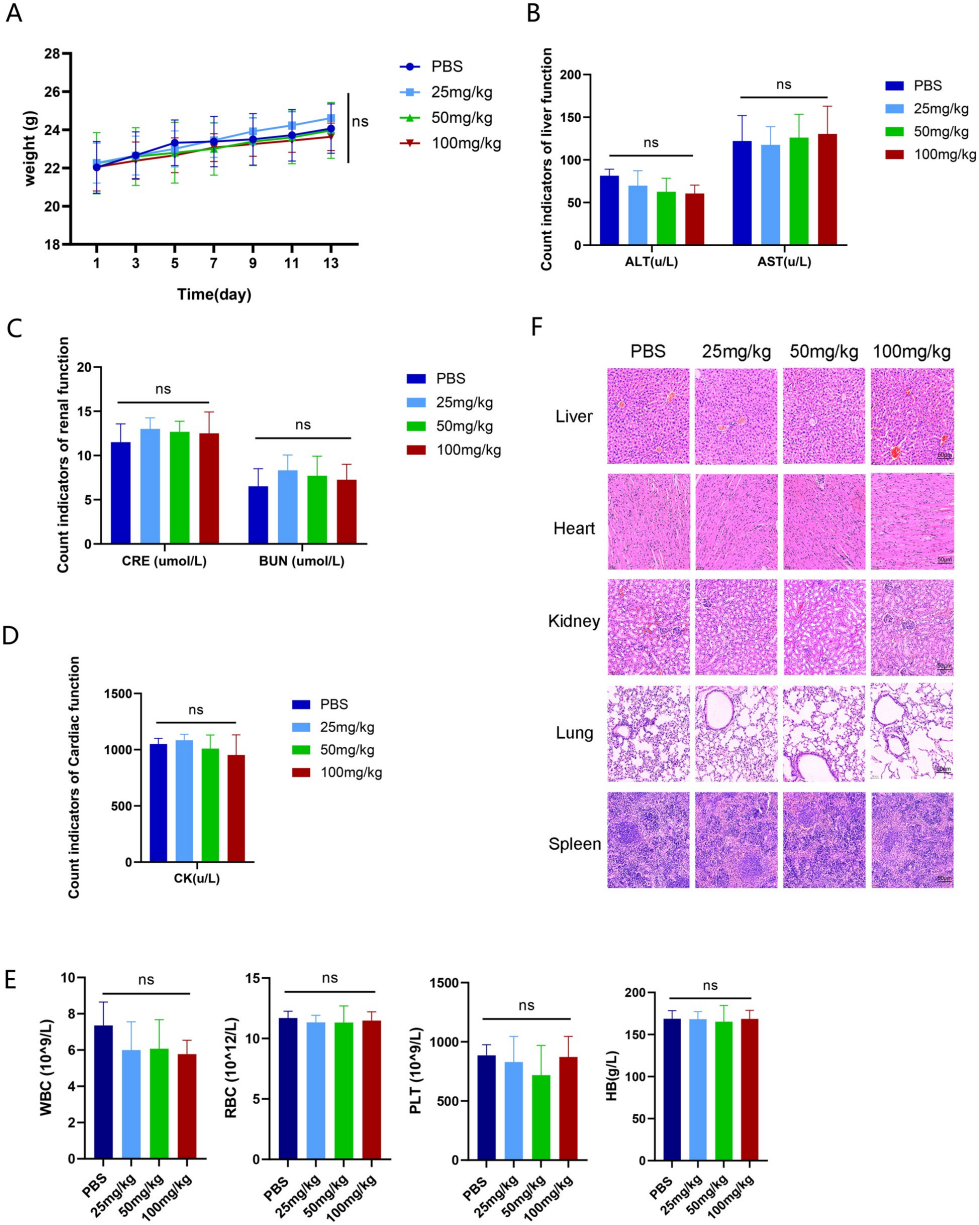
In vivo safety of ZSTK474 (A)The Body weight of nude mice (each group, n = 6). (B-D) Liver, kidney, and cardiac injury function markers of nude mice (each group, n = 6). (E) Complete blood cell counts of nude mice (each group, n = 6). (F). HE staining of major organ (each group, n = 6), scale bar: 50μm. ns P> 0.05.

The safety of ZSTK474 was assessed in the WiT49 tumor mouse model. Comparison of post-administration body weight changes with the PBS control group in tumor bearing mice indicated no significant differences (Fig 3A), affirming its safety profile. Furthermore, no notable variances were observed in hematological indices and organ pathology examination between the ZSTK474-treated group and the control group (Fig 3B–3F and Table 3).

**Table 3:** Results of laboratory examination in each group.

	PBS	25mg/kg ZSTK474	50mg/kg ZSTK474	100mg/kg ZSTK474	P
ALT	81.4(70.7–90.2)	69.7(44.7–96.9)	62.6(48.1–88.2)	60.6(50.4–76.5)	0.055
AST	122.1 (101.3–177.9)	117.5 (95.8–153.7)	126.1 (99.4–174.1)	130.4 (97.7–173.1)	0.229
CRE	11.5(8.0–13.0)	13.0(11.0–15.0)	12.7(11.0–14.0)	12.5(10.0–15.0)	0.754
BUN	6.5(4.7–10.1)	8.3(5.6–10.6)	7.7(5.1–10.8)	7.3(5.1–10.1)	0.457
CK	1051.0 (995.0–1105.0)	1084.2 (1029.0–1145.0)	1009.5 (793.0–1141.0)	952.5 (637.0–1135.0)	0.259

ALT: Alanine transaminase;AST: Aspartate aminotransferase; CRE: Serum creatinine;BUN: Blood urea nitrogen; LDH: Lactate dehydrogenase; CK: Creatine kinase.

### 3.4. Anti-tumor effect of ZSTK474 in vivo

**Fig 4:**
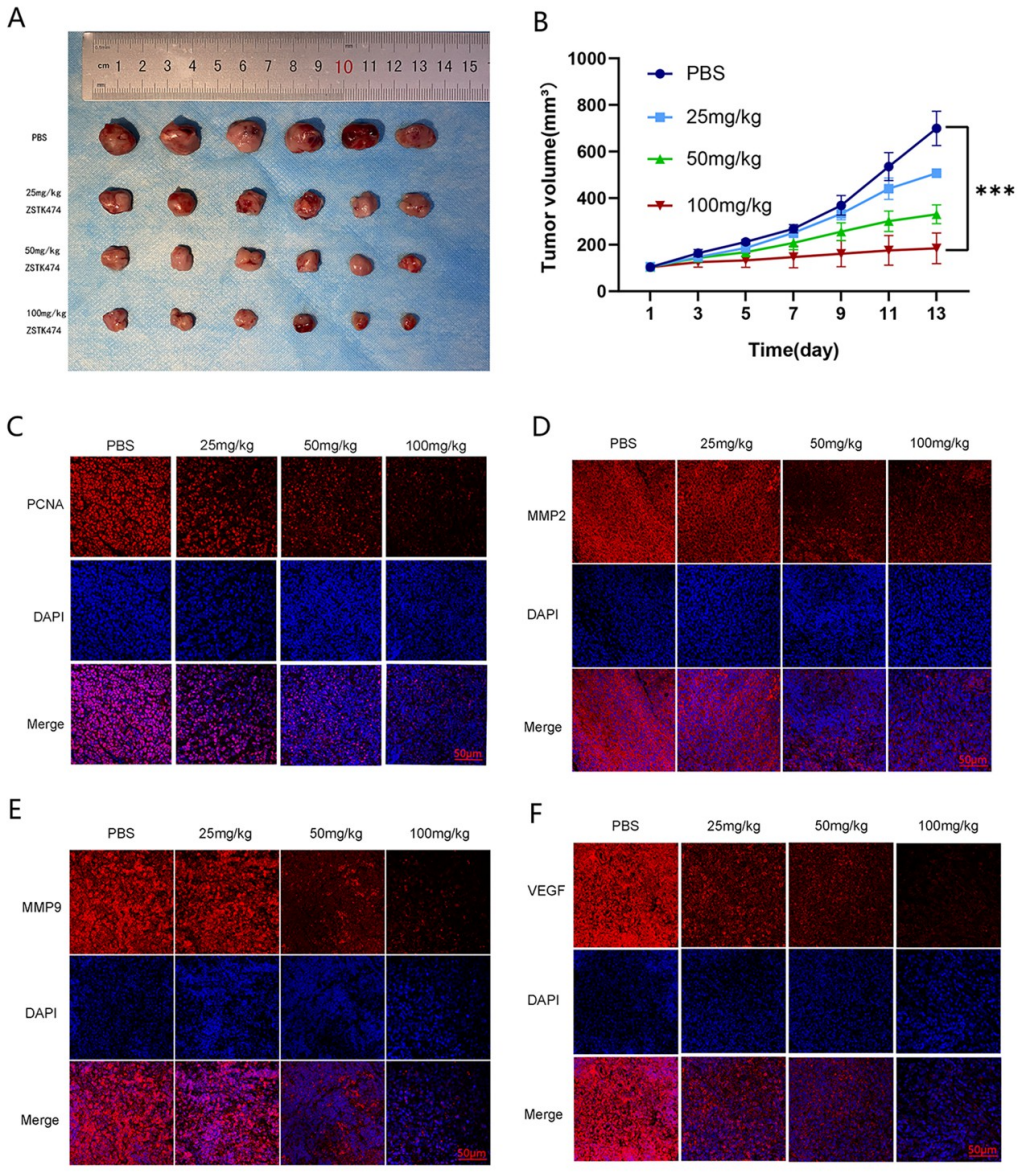
In vivo therapeutic effect of ZSTK474 (each group, n = 6). (A) Tumor tissues (each group, n = 6). (B) Trends in tumor volume growth (each group, n = 6). (C-F), Immunofluorescence of tumor (each group, n = 6), scale bars: 50μm.

A notable trend was observed during the monitoring of subcutaneous tumor volume. On day 5 of the intervention, the high-dose treatment group exhibited initial reductions in tumor volume relative to the control group, with further decreases observed over time. On day 11, there was a notable reduction in tumor volumes in the treatment group compared to the control group (Fig 4A and 4B). Immunofluorescence analysis indicated decreased levels of markers related to proliferation, invasion, migration, and angiogenesis in tumor tissues treated with ZSTK474 at doses of 25, 50, and 100 mg/kg. Notably, levels of PCNA, MMP2, MMP9, and VEGF were markedly reduced (Fig 4C–4F).

### 3.5. Inhibition of proliferation, migration, and invasion of WT cells by ZSTK474

**Fig 5:**
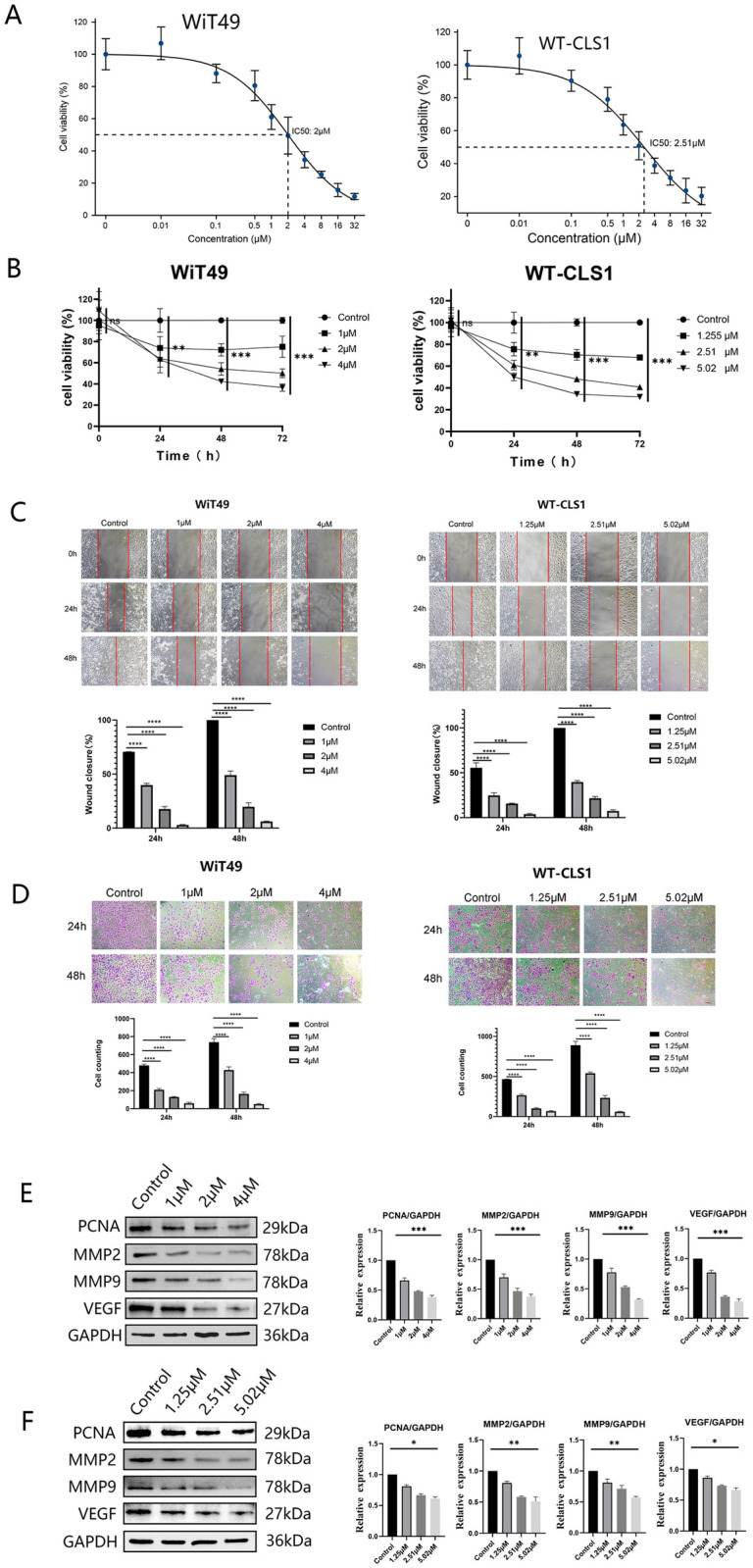
ZSTK474 inhibits the proliferation, migration, and invasion of WT cells. (A) The ZSTK474 IC50 to WiT49 and WT-CLS1. (B) Proliferation curve of WiT49 and WT-CLS1. (C) Migration ability of WiT49 and WT-CLS1. (D) Invasion ability of WiT49 and WT-CLS1. (E-F) The expression of proliferation, migration, and invasion-related protein of WT cells. * P < 0.05, ** P < 0.01, *** P < 0.001.

The in vitro investigation examined the effect of ZSTK474 on WT cells. Following a 48-hour treatment period, IC50 values of 2 μM and 2.51 μM were determined for WiT49 cells and WT-CLS1 cells, respectively (Fig 5A). Subsequent analyses demonstrated a dose-dependent suppression of cell growth by ZSTK474 (Fig 5B). Scratch assays indicated reduced migration rates, confirming the inhibitory effect of ZSTK474 on cell migration (Fig 5C). Transwell assays further validated a decrease in invasion capacity (Fig 5D). Western blotting revealed lowered expression levels of PCNA, MMP2, MMP9, and VEGF, providing additional evidence for the anti-tumor efficacy of ZSTK474 (Fig 5E and 5F).

### 3.6. ZSTK474 promotes apoptosis and induces G0/G1 arrest

**Fig 6:**
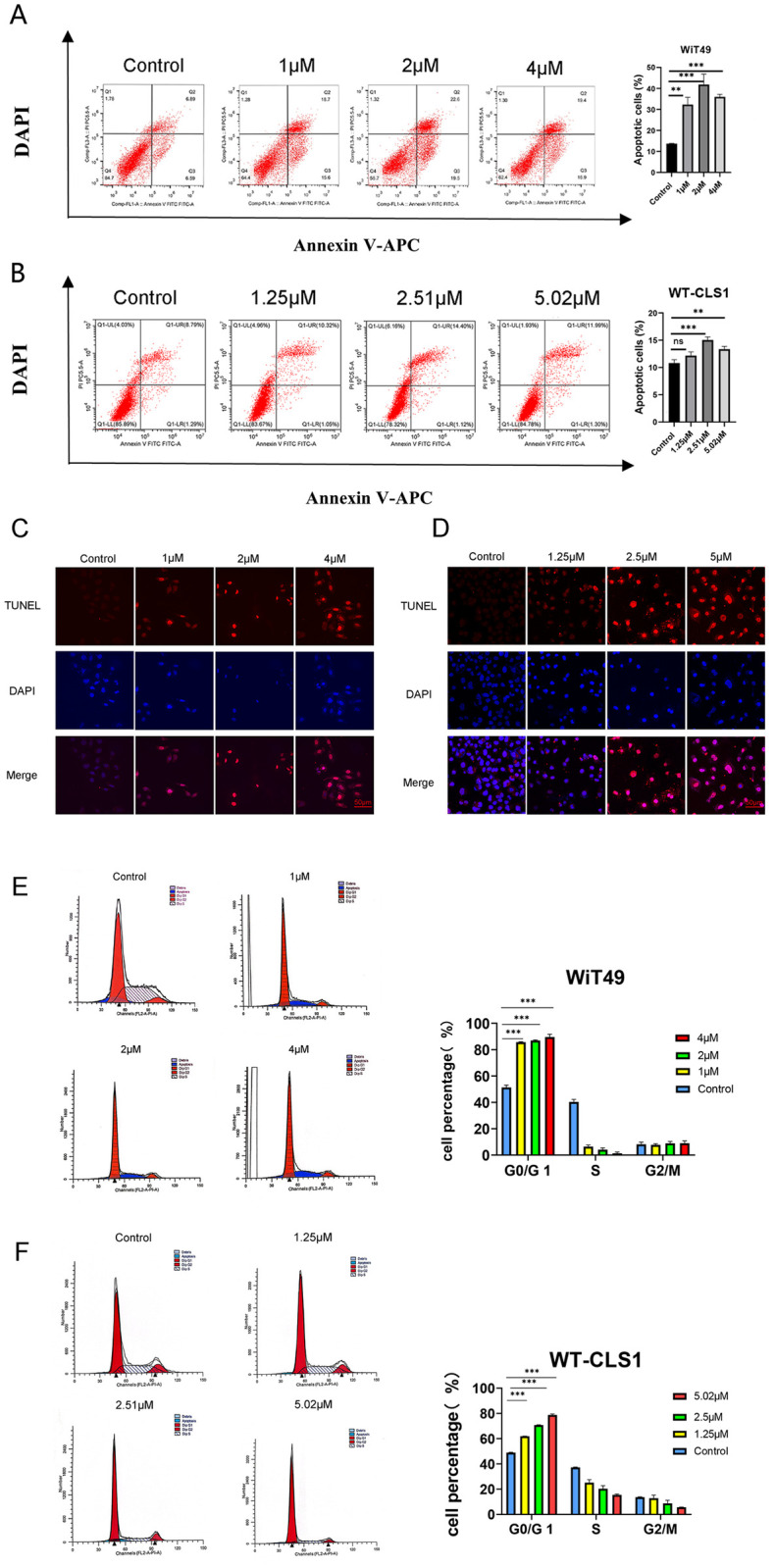
ZSTK474 induces apoptosis and G0/G1 cell phrase arrest. (A-B) Annexin V apoptosis assay in WiT49 and WT-CLS1. (C-D) TUNEL of WiT49 and WT-CLS1, scale bar:50μm. (E-F) The cell cycle of WiT49 and WT-CLS1.

Following the utilization of the Annexin-V Apoptosis Kit, a notable increase in apoptosis was observed in the treated cells. Specifically, WiT49 cells exhibited heightened sensitivity, with a 20% rise in apoptotic cells compared to the controls after 48 hours of treatment at the IC50 dose, whereas WT-CLS1 cells maintained levels below 10% (Fig 6A and 6B). Subsequent TUNEL assay validation confirmed late apoptotic DNA damage response in the treated cells (Fig 6C and 6D). Flow cytometry assays demonstrated a significant rise in the percentage of cells in the G0/G1 phase in WT cells after exposure to different concentrations of ZSTK474 (Fig 6E and 6F).

### 3.7. Cell cycle arrest in the G0/G1 phase induced by inhibition of the PI3K-Akt pathway

**Fig 7:**
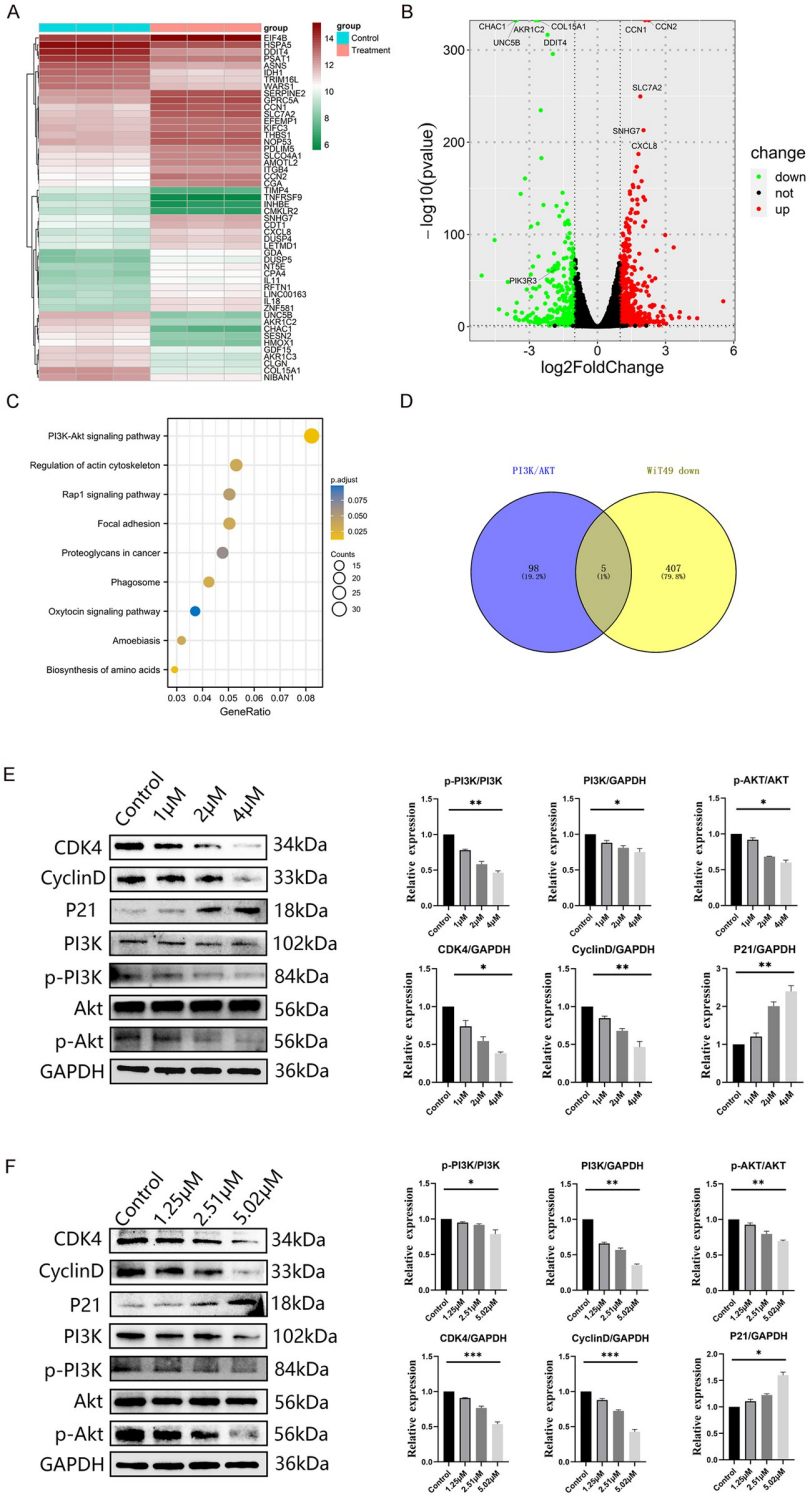
RNA-seq, bioinformatics analysis of treated WiT49, and PI3K/Akt signaling pathway detection. (A) Heatmap, (B) Volcano map, (C) KEGG, (D) Venn diagram, (E-F) WB of WiT49 and WT-CLS1. * P<0.05, ** P<0.01, *** P<0.001.

Following transcriptome sequencing of ZSTK474-treated WiT49 cells, 1069 DEGs were identified, including 412 downregulated and 657 upregulated genes (Fig 7A and 7B). The KEGG analysis indicated a substantial enrichment of these genes in the PI3K/Akt pathway (Fig 7C). Specifically, five genes, including CDKN1A, FGF18, NRG4, PDGFRB and PIK3R3, were found to be common among the down-regulated genes and the PI3K/Akt pathway, indicating their potential role in cell cycle regulation (Fig 7D). Subsequent protein analysis validated the suppressing role of ZSTK474 on the PI3K/Akt pathway, leading to a reduction in cyclin D and CDK4 levels and an elevation in P21 expression (Fig 7E and 7F).

### 3.8. ZSTK474 inhibits cell cycle progression by targeting PIK3R3

**Fig 8:**
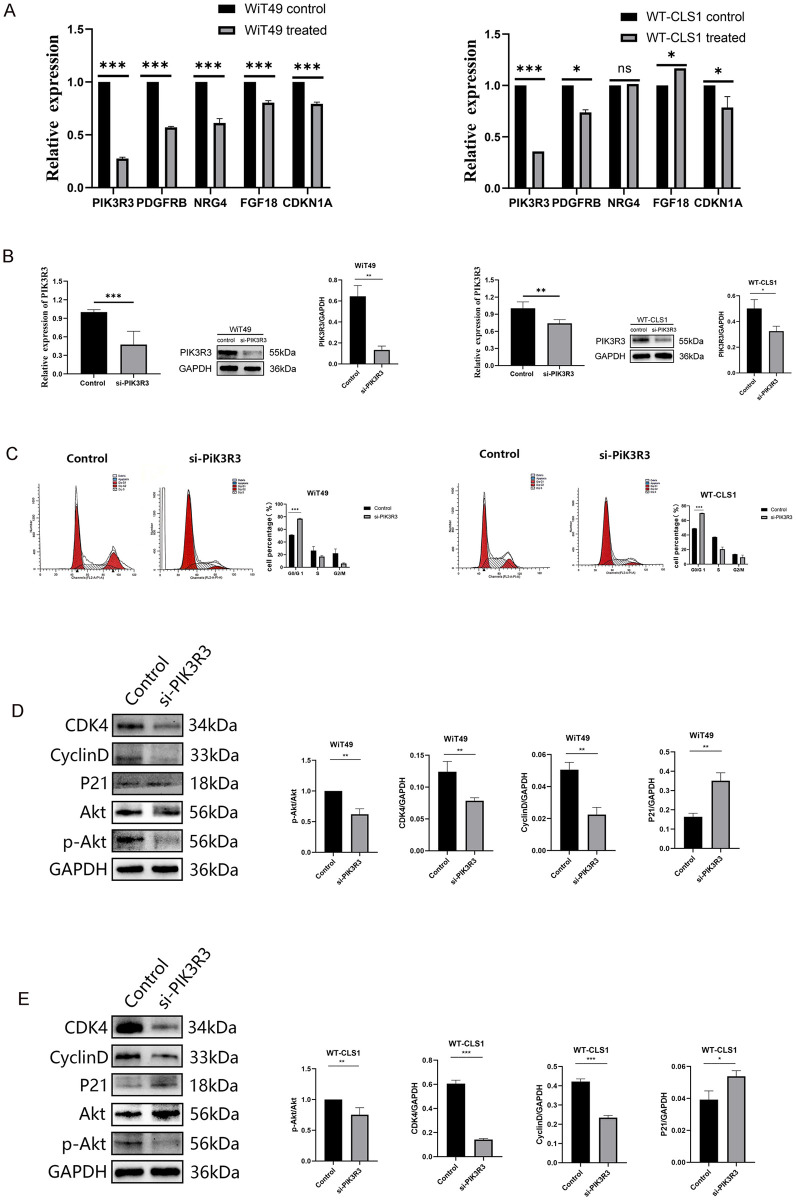
ZSTK474 targeting PIK3R3 induces G0/G1 cell cycle block by inhibiting PI3K/Akt signaling pathway in WT. (A) The mRNA expression of PIK3R3 after ZSTK474 intervention in WiT49 and WT-CLS1. (B) The mRNA expression and protein expression of si-PIK3R3 transfection in WiT49 and WT-CLS1. (C) The cell cycle of si-PIK3R3 transfection in WiT49 and WT-CLS1. (D-E) WB of si-PIK3R3 transfection in WiT49 and WT-CLS1.

To assess the impact of ZSTK474, we examined the expression of five genes in WT cells post-ZSTK474 treatment using RT-PCR. Our results indicated a significant decrease in RNA levels of all five genes in ZSTK474-treated WiT49 cells, with PIK3R3 showing the most pronounced downregulation in WT-CLS1 cells (Fig 8A). This suggests that ZSTK474 primarily targets the inhibition of PIK3R3. To validate the observations mentioned above, RNA interference was employed to suppress PIK3R3 expression in WT cells (Fig 8B). This manipulation led to reduced levels of PIK3R3 at both transcriptional and translational levels, thereby causing cell cycle arrest in the G0/G1 phase (Fig 8C). Furthermore, PIK3R3 inhibition led to diminished Akt phosphorylation, indicating suppression of the PI3K/Akt pathway, and affected expressions of cell cycle regulators, including increased P21 and decreased cyclin D and CDK4 expression (Fig 8D–8E).

## 4. Discussion

The management of tumors has historically posed a significant challenge in the medical field. However, the advent of targeted drug therapy, which focuses on specific signaling pathways, has instilled new hope for cancer treatment. By utilizing clinical data and RNA-Seq information from the TARGET-WT database, this study investigated and identified DEGs between nephroblastoma and paraneoplastic tissues. Our findings revealed 3170 DEGs, among which 101 genes exhibited upregulation and correlated with poor prognosis. Subsequent investigation of these 101 genes through the CMap drug database pinpointed ZSTK474 as a promising targeted therapeutic candidate for nephroblastoma. The dysregulated PI3K/Akt pathway is associated with various malignancies, playing a pivotal role in aberrant cellular processes, for example, apoptosis, invasion, metastasis, proliferation, angiogenesis, cell cycle regulation, and resistance to chemotherapy [36–41]. Consequently, it is postulated that nephroblastoma may exhibit heightened activity in the PI3K/Akt pathway, suggesting potential therapeutic benefits from targeting this pathway with specific pharmaceutical agents.

We first examined the activity of the PI3K/Akt pathway in nephroblastoma to assess its suitability for targeted treatment with ZSTK474. By analyzing the TARGET-WT dataset, we identified DEGs and performed enrichment analysis, revealing a predominant enrichment of DEGs in the PI3K/Akt pathway. Subsequently, our sequencing data analysis from eight human nephroblastoma samples with differential gene expression showed enrichment in the cell cycle and PI3K/Akt pathways. Additionally, Western blot analyses confirmed significantly elevated expressions of p-Akt/Akt and p-PI3K/PI3K in WT tissues and cells, aligning with existing literature indicating dysfunction of the PI3K/Akt pathway in nephroblastoma [42, 43].

A subcutaneous xenograft mouse model utilizing the WiT49 anaplastic Wilms tumor cell line was established to evaluate the anti-tumor efficacy and safety of ZSTK474 in vivo, given the unfavorable prognosis associated with anaplastic Wilms tumor in pediatric patients. Previous animal studies have indicated a favorable safety profile for ZSTK474, demonstrating minimal toxicity [27, 28, 30, 44]. In our investigation, varying doses of ZSTK474 were orally subjected to nude mice, with no notable weight loss observed. Comprehensive evaluations, including blood tests, liver and kidney function assessments, and cardiac enzyme analyses, conducted after two weeks of drug administration revealed no significant indications of harm. Histomorphological analysis of major organs via HE staining exhibited no significant variances across experimental groups. Acknowledging the potential for organ and bone marrow hematopoietic system impairment to accrue over time is crucial. Consequently, our findings suggest that short-term oral ZSTK474 administration is safe and viable. The literature indicates that chronic oral administration of ZSTK474 at a dose of 800 mg/kg for more than 28 days did not result in any dermal toxicity. The most significant side effect observed was a weight loss of 14% by day 28, which remained within the tolerable range, and there was no evidence of toxic damage to vital organs [30]. Further extensive investigations are required to evaluate the potential long-term risks of administering ZSTK474.

Our study on the anti-tumor effects of ZSTK474 demonstrated a significant inhibition of tumor growth in vivo, with a dose-dependent trend. This finding was drawn from our assessment of subcutaneous tumor dimensions, volume, and excised tumor weight in nude mice. Additionally, our immunofluorescence assay of subcutaneous tumor tissues indicated that ZSTK474 effectively suppressed the expressions of vascular endothelial growth factor VEGF and matrix metalloproteinases MMP2 and MMP9. These results align with previous studies by Zhou [33] and Baiz et al. [31] supporting the efficacy of ZSTK474 in impeding tumor invasion and metastasis. Furthermore, our analysis of the proliferation-related marker PCNA revealed a dose-dependent decrease in its expression with increasing levels of drug intervention, providing further evidence of ZSTK474’s anti-tumor properties in vivo.

Our in vitro cellular experiments confirmed the efficacy of ZSTK474 in inhibiting the proliferation, migration, and invasion of WT cells. Furthermore, ZSTK474 demonstrated the ability to trigger apoptosis in WT cells, albeit at a lower rate than anticipated after 48 hours. Specifically, the apoptosis rate in WT-CLS1 cells was below 10%, while WiT49 cells exhibited a higher rate of 20%. These results suggest that ZSTK474 can induce apoptosis in both WiT49 and WT-CLS1 cells, with variations in the apoptotic response between the two cell types. Prior research has shown inconsistent levels of ZSTK474-induced apoptosis, indicating a potential reliance on cell type [30, 45]. Additionally, positive outcomes from the TUNEL assay revealed a delayed DNA damage response post ZSTK474 treatment, implying that the observed apoptotic effects may not entirely account for the significant inhibitory influence of ZSTK474 on WT cells, as evidenced in our investigation.

Previous studies have shown that ZSTK474 induces cell cycle arrest in the G0/G1 phase [27, 33]. This study examined the role of ZSTK474 on the nephroblastoma cell cycle through flow cytometry and the results confirmed a G0/G1 phase arrest induced by ZSTK474. Subsequent analysis of protein markers revealed the downregulation of CDK4 and Cyclin D, upregulation of P21, and reduced levels of p-PI3K/PI3K and p-Akt/Akt in WT cells, which align with previous research [31, 33, 46]. Therefore, these findings collectively suggest that ZSTK474 induces cell cycle arrest by effectively inhibiting the PI3K/Akt pathway, thereby suppressing the proliferation, invasion, and migration of nephroblastoma cells.

PIK3R3, a PI3K regulatory domain P85 gene family member, is crucial for activating downstream signaling pathways through phosphorylated Akt, facilitating cellular processes such as survival, epithelial-mesenchymal transition, and cancer stem cell generation [47]. Elevated expression of PIK3R3 regulatory subunits has been observed across various cancer types, playing a pivotal role in tumor advancement and cell growth [47–49]. It has been demonstrated that the manipulation of the PI3K/Akt pathway via PIK3R3 influences the cell cycle, as inhibiting or reducing PIK3R3 leads to G0/G1 phase arrest [50]. In our research, flow cell cycle analysis was conducted to examine the influence of PIK3R3 siRNA transfection on the cell cycle. The results indicated transfection with PIK3R3 siRNA markedly arrested cells in the G0/G1 phase. This transfection increased P21 expression and decreased CDK4 and Cyclin D levels. Furthermore, PIK3R3 siRNA transfection led to reduced p-Akt expression. Our study unveiled that ZSTK474-mediated inhibition of PIK3R3 reduced p-Akt levels, leading to significant G0/G1 phase arrest, enhanced P21 activity, and repression of Cyclin D and CDK4 functions. Collectively, ZSTK474’s targeting of PIK3R3 induces G0/G1 phase arrest in cells by suppressing the PI3K/Akt signaling pathway, ultimately hindering the progression of nephroblastoma.

Our findings suggest that ZSTK474 effectively targets PIK3R3 to inhibit WT, positioning it as a promising candidate for novel anticancer therapies. However, this study has several limitations. Firstly, we do not have long-term safety data for ZSTK474 in living organisms. Extended safety studies are needed to assess the drug’s impact over time and ensure whether adverse effects arise from prolonged exposure. Furthermore, future research should explore the potential for tumor relapse or the development of resistance following the cessation of treatment. Additionally, the safety and efficacy of re-administering ZSTK474 to patients who have previously undergone treatment with the drug warrant further investigation. Given the ubiquitous presence of PI3K in various normal tissues, comprehensive preclinical investigations are imperative. These studies should encompass molecular pharmacology, pharmacokinetics, and cytotoxicity assessments to ascertain whether ZSTK474 induces unacceptable off-target effects. Future research may concentrate on developing engineered extracellular vesicles to augment the efficacy of ZSTK474 while mitigating systemic toxicity.

## 5. Conclusion

In summary, our research establishes the efficacy and safety of ZSTK474 in inhibiting nephroblastoma cell proliferation, invasion, and migration. Moreover, ZSTK474 potentially hinders cancer progression by disrupting the PI3K/Akt pathway, resulting in G0/G1 cell cycle arrest primarily through targeting PIK3R3. These results offer a compelling rationale for further investigating ZSTK474 and other PI3K inhibitors as promising therapeutic options for nephroblastoma management.

## Supporting information

S1 File The RNA-seq analysis of ZSTK474 treated WiT49. (CSV)

S2 File The raw images of western blot. (ZIP)

## References

[1] Balis F, Green DM, Anderson C, Cook S, Dhillon J, Gow K, et al. Wilms Tumor (Nephroblastoma), Version 2.2021, NCCN Clinical Practice Guidelines in Oncology. J Natl Compr Canc Netw. 2021 Aug 1;19(8):945–977. doi: 10.6004/jnccn.2021.0037 34416707

[2] Spreafico F, Fernandez CV, Brok J, Nakata K, Vujanic G, Geller JI, et al. Wilms tumour. Nat Rev Dis Primers. 2021 Oct 14;7(1):75. doi: 10.1038/s41572-021-00308-8 34650095

[3] Irtan S, Ehrlich PF, Pritchard-Jones K. Wilms tumor: "State-of-the-art" update, 2016. Semin Pediatr Surg. 2016 Oct;25(5):250–256. doi: 10.1053/j.sempedsurg.2016.09.003 27955727

[4] Gratias EJ, Dome JS, Jennings LJ, Chi YY, Tian J, Anderson J, et al. Association of Chromosome 1q Gain With Inferior Survival in Favorable-Histology Wilms Tumor: A Report From the Children’s Oncology Group. J Clin Oncol. 2016 Sep 10;34(26):3189–94. doi: 10.1200/JCO.2015.66.1140 27400937 PMC5012705

[5] Dome JS, Graf N, Geller JI, Fernandez CV, Mullen EA, Spreafico F, et al. Advances in Wilms Tumor Treatment and Biology: Progress Through International Collaboration. J Clin Oncol. 2015 Sep 20;33(27):2999–3007. doi: 10.1200/JCO.2015.62.1888 26304882 PMC4567702

[6] Dome JS, Cotton CA, Perlman EJ, Breslow NE, Kalapurakal JA, Ritchey ML, et al. Treatment of anaplastic histology Wilms’ tumor: results from the fifth National Wilms’ Tumor Study. J Clin Oncol. 2006 May 20;24(15):2352–8. doi: 10.1200/JCO.2005.04.7852 16710034

[7] Groenendijk A, Spreafico F, de Krijger RR, Drost J, Brok J, Perotti D, et al. Prognostic Factors for Wilms Tumor Recurrence: A Review of the Literature. Cancers (Basel). 2021 Jun 23;13(13):3142. doi: 10.3390/cancers13133142 34201787 PMC8268923

[8] Evageliou N, Renfro LA, Geller J, Perlman E, Kalapurakal J, Paulino A, et al. Prognostic impact of lymph node involvement and loss of heterozygosity of 1p or 16q in stage III favorable histology Wilms tumor: A report from Children’s Oncology Group Studies AREN03B2 and AREN0532. Cancer. 2024 Mar 1;130(5):792–802. doi: 10.1002/cncr.35084 37902955 PMC10993001

[9] Saltzman AF, Cost NG, Romao RLP. Wilms Tumor. Urol Clin North Am. 2023 Aug;50(3):455–464. doi: 10.1016/j.ucl.2023.04.008 37385707

[10] Madanat-Harjuoja LM, Renfro LA, Klega K, Tornwall B, Thorner AR, Nag A, et al. Circulating Tumor DNA as a Biomarker in Patients With Stage III and IV Wilms Tumor: Analysis From a Children’s Oncology Group Trial, AREN0533. J Clin Oncol. 2022 Sep 10;40(26):3047–3056. doi: 10.1200/JCO.22.00098 35580298 PMC9462535

[11] Murphy AJ, Brzezinski J, Renfro LA, Tornwall B, Malek MM, Benedetti DJ, et al. Long-term outcomes and patterns of relapse in patients with bilateral Wilms tumor or bilaterally predisposed unilateral Wilms tumor, a report from the COG AREN0534 study. Int J Cancer. 2024 Jul 8. doi: 10.1002/ijc.35080 38973574 PMC11570340

[12] Verschuur A, Van Tinteren H, Graf N, Bergeron C, Sandstedt B, de Kraker J. Treatment of pulmonary metastases in children with stage IV nephroblastoma with risk-based use of pulmonary radiotherapy. J Clin Oncol. 2012 Oct 1;30(28):3533–9. doi: 10.1200/JCO.2011.35.8747 22927531

[13] Spreafico F, Pritchard Jones K, Malogolowkin MH, Bergeron C, Hale J, de Kraker J, et al. Treatment of relapsed Wilms tumors: lessons learned. Expert Rev Anticancer Ther. 2009 Dec;9(12):1807–15. doi: 10.1586/era.09.159 19954292

[14] Malogolowkin M, Spreafico F, Dome JS, van Tinteren H, Pritchard-Jones K, van den Heuvel-Eibrink MM, et al. COG Renal Tumors Committee and the SIOP Renal Tumor Study Group. Incidence and outcomes of patients with late recurrence of Wilms’ tumor. Pediatr Blood Cancer. 2013 Oct;60(10):1612–5. doi: 10.1002/pbc.24604 23737480

[15] Maschietto M, Piccoli FS, Costa CM, Camargo LP, Neves JI, Grundy PE, et al. Gene expression analysis of blastemal component reveals genes associated with relapse mechanism in Wilms tumour. Eur J Cancer. 2011 Dec;47(18):2715–22. doi: 10.1016/j.ejca.2011.05.024 21703850

[16] Paulino AC, Wen BC, Brown CK, Tannous R, Mayr NA, Zhen WK, et al. Late effects in children treated with radiation therapy for Wilms’ tumor. Int J Radiat Oncol Biol Phys. 2000 Mar 15;46(5):1239–46. doi: 10.1016/s0360-3016(99)00534-9 10725637

[17] Ehrlich P, Chi YY, Chintagumpala MM, Hoffer FA, Perlman EJ, Kalapurakal JA, et al. Results of the First Prospective Multi-institutional Treatment Study in Children With Bilateral Wilms Tumor (AREN0534): A Report From the Children’s Oncology Group. Ann Surg. 2017 Sep;266(3):470–478. doi: 10.1097/SLA.0000000000002356 28795993 PMC5629006

[18] Dix DB, Seibel NL, Chi YY, Khanna G, Gratias E, Anderson JR, et al. Treatment of Stage IV Favorable Histology Wilms Tumor With Lung Metastases: A Report From the Children’s Oncology Group AREN0533 Study. J Clin Oncol. 2018 Jun 1;36(16):1564–1570. doi: 10.1200/JCO.2017.77.1931 29659330 PMC6075846

[19] Alam MS, Sultana A, Sun H, Wu J, Guo F, Li Q, et al. Bioinformatics and network-based screening and discovery of potential molecular targets and small molecular drugs for breast cancer. Front Pharmacol. 2022 Sep 20;13:942126. doi: 10.3389/fphar.2022.942126 36204232 PMC9531711

[20] Palmisani F, Kovar H, Kager L, Amann G, Metzelder M, Bergmann M. Systematic review of the immunological landscape of Wilms tumors. Mol Ther Oncolytics. 2021 Jul 13;22:454–467. doi: 10.1016/j.omto.2021.06.016 . 34553032 PMC8430048

[21] Hong B, Dong R. Research advances in the targeted therapy and immunotherapy of Wilms tumor: a narrative review. Transl Cancer Res. 2021 Mar;10(3):1559–1567. doi: 10.21037/tcr-20-3302 35116480 PMC8799117

[22] Tian XM, Xiang B, Jin LM, Mi T, Wang JK, Zhanghuang C, et al. Immune-related gene signature associates with immune landscape and predicts prognosis accurately in patients with Wilms tumour. Front Immunol. 2022 Sep 12;13:920666. doi: 10.3389/fimmu.2022.920666 36172369 PMC9510599

[23] Lamb J. The Connectivity Map: a new tool for biomedical research. Nat Rev Cancer. 2007 Jan;7(1):54–60. doi: 10.1038/nrc2044 17186018

[24] Lamb J, Crawford ED, Peck D, Modell JW, Blat IC, Wrobel MJ, et al. The Connectivity Map: using gene-expression signatures to connect small molecules, genes, and disease. Science. 2006 Sep 29;313(5795):1929–35. doi: 10.1126/science.1132939 17008526

[25] Zhao Y, Chen X, Chen J, Qi X. Decoding Connectivity Map-based drug repurposing for oncotherapy. Brief Bioinform. 2023 May 19;24(3):bbad142. doi: 10.1093/bib/bbad142 37068308

[26] Kong DX, Yamori T. ZSTK474, a novel phosphatidylinositol 3-kinase inhibitor identified using the JFCR39 drug discovery system. Acta Pharmacol Sin. 2010 Sep;31(9):1189–97. doi: 10.1038/aps.2010.150 20729870 PMC4002321

[27] Dan S, Yoshimi H, Okamura M, Mukai Y, Yamori T. Inhibition of PI3K by ZSTK474 suppressed tumor growth not via apoptosis but G0/G1 arrest. Biochem Biophys Res Commun. 2009 Jan 30;379(1):104–9. doi: 10.1016/j.bbrc.2008.12.015 19094964

[28] Kong D, Okamura M, Yoshimi H, Yamori T. Antiangiogenic effect of ZSTK474, a novel phosphatidylinositol 3-kinase inhibitor. Eur J Cancer. 2009 Mar;45(5):857–65. doi: 10.1016/j.ejca.2008.12.007 19144509

[29] Liu J, Tan X, Zhao W, Liu J, Xing X, Fan G, et al. In Vitro and In Vivo Antimetastatic Effects of ZSTK474 on Prostate Cancer DU145 Cells. Curr Cancer Drug Targets. 2019;19(4):321–329. doi: 10.2174/1568009618666180911101310 30205797

[30] Yaguchi S, Fukui Y, Koshimizu I, Yoshimi H, Matsuno T, Gouda H, et al. Antitumor activity of ZSTK474, a new phosphatidylinositol 3-kinase inhibitor. J Natl Cancer Inst. 2006 Apr 19;98(8):545–56. doi: 10.1093/jnci/djj133 16622124

[31] Baiz D, Hassan S, Choi YA, Flores A, Karpova Y, Yancey D, et al. Combination of the PI3K inhibitor ZSTK474 with a PSMA-targeted immunotoxin accelerates apoptosis and regression of prostate cancer. Neoplasia. 2013 Oct;15(10):1172–83. doi: 10.1593/neo.13986 24204196 PMC3819633

[32] Dan S, Okamura M, Mukai Y, Yoshimi H, Inoue Y, Hanyu A, et al. ZSTK474, a specific phosphatidylinositol 3-kinase inhibitor, induces G1 arrest of the cell cycle in vivo. Eur J Cancer. 2012 Apr;48(6):936–43. doi: 10.1016/j.ejca.2011.10.006 22088482

[33] Zhou Q, Chen Y, Chen X, Zhao W, Zhong Y, Wang R, et al. In Vitro Antileukemia Activity of ZSTK474 on K562 and Multidrug Resistant K562/A02 Cells. Int J Biol Sci. 2016 Apr 8;12(6):631–8. doi: 10.7150/ijbs.14878 27194941 PMC4870707

[34] Lin L, Gaut D, Hu K, Yan H, Yin D, Koeffler HP. Dual targeting of glioblastoma multiforme with a proteasome inhibitor (Velcade) and a phosphatidylinositol 3-kinase inhibitor (ZSTK474). Int J Oncol. 2014 Feb;44(2):557–62. doi: 10.3892/ijo.2013.2205 24297065 PMC3898871

[35] Mengelbier LH, Bexell D, Sehic D, Ciornei CD, Gisselsson D. Orthotopic Wilms tumor xenografts derived from cell lines reflect limited aspects of tumor morphology and clinical characteristics. Pediatr Blood Cancer. 2014 Nov;61(11):1949–54. doi: 10.1002/pbc.25131 25044705

[36] Janku F, Yap TA, Meric-Bernstam F. Targeting the PI3K pathway in cancer: are we making headway? Nat Rev Clin Oncol. 2018 May;15(5):273–291. doi: 10.1038/nrclinonc.2018.28 29508857

[37] Hanker AB, Kaklamani V, Arteaga CL. Challenges for the Clinical Development of PI3K Inhibitors: Strategies to Improve Their Impact in Solid Tumors. Cancer Discov. 2019 Apr;9(4):482–491. doi: 10.1158/2159-8290.CD-18-1175 30867161 PMC6445714

[38] Fattahi S, Amjadi-Moheb F, Tabaripour R, Ashrafi GH, Akhavan-Niaki H. PI3K/AKT/mTOR signaling in gastric cancer: Epigenetics and beyond. Life Sci. 2020 Dec 1;262:118513. doi: 10.1016/j.lfs.2020.118513 33011222

[39] Alam J, Huda MN, Tackett AJ, Miah S. Oncogenic signaling-mediated regulation of chromatin during tumorigenesis. Cancer Metastasis Rev. 2023 Jun;42(2):409–425. doi: 10.1007/s10555-023-10104-3 37147457 PMC10348982

[40] Ippen FM, Grosch JK, Subramanian M, Kuter BM, Liederer BM, Plise EG, et al. Targeting the PI3K/Akt/mTOR pathway with the pan-Akt inhibitor GDC-0068 in PIK3CA-mutant breast cancer brain metastases. Neuro Oncol. 2019 Nov 4;21(11):1401–1411. doi: 10.1093/neuonc/noz105 . 31173106 PMC6827829

[41] Ediriweera MK, Tennekoon KH, Samarakoon SR. Role of the PI3K/AKT/mTOR signaling pathway in ovarian cancer: Biological and therapeutic significance. Semin Cancer Biol. 2019 Dec;59:147–160. doi: 10.1016/j.semcancer.2019.05.012 31128298

[42] Polosukhina D, Love HD, Correa H, Su Z, Dahlman KB, Pao W, et al. Functional KRAS mutations and a potential role for PI3K/AKT activation in Wilms tumors. Mol Oncol. 2017 Apr;11(4):405–421. doi: 10.1002/1878-0261.12044 28188683 PMC5378659

[43] Luo X, Dong J, He X, Shen L, Long C, Liu F, et al. MiR-155-5p exerts tumor-suppressing functions in Wilms tumor by targeting IGF2 via the PI3K signaling pathway. Biomed Pharmacother. 2020 May;125:109880. doi: 10.1016/j.biopha.2020.109880 32004974

[44] Wang P, He Y, Li D, Han R, Liu G, Kong D, et al. Class I PI3K inhibitor ZSTK474 mediates a shift in microglial/macrophage phenotype and inhibits inflammatory response in mice with cerebral ischemia/reperfusion injury. J Neuroinflammation. 2016 Aug 22;13(1):192. doi: 10.1186/s12974-016-0660-1 27549161 PMC4994222

[45] Isoyama S, Tamaki N, Noguchi Y, Okamura M, Yoshimatsu Y, Kondo T, et al. Subtype-selective induction of apoptosis in translocation-related sarcoma cells induced by PUMA and BIM upon treatment with pan-PI3K inhibitors. Cell Death Dis. 2023 Feb 27;14(2):169. doi: 10.1038/s41419-023-05690-7 36849535 PMC9971170

[46] Zhao W, Guo W, Zhou Q, Ma SN, Wang R, Qiu Y, et al. In vitro antimetastatic effect of phosphatidylinositol 3-kinase inhibitor ZSTK474 on prostate cancer PC3 cells. Int J Mol Sci. 2013 Jun 28;14(7):13577–91. doi: 10.3390/ijms140713577 23812078 PMC3742204

[47] Wang G, Yang X, Li C, Cao X, Luo X, Hu J. PIK3R3 induces epithelial-to-mesenchymal transition and promotes metastasis in colorectal cancer. Mol Cancer Ther. 2014 Jul;13(7):1837–47. doi: 10.1158/1535-7163.MCT-14-0049 24837077

[48] Hu J, Xia X, Cheng A, Wang G, Luo X, Reed MF, et al. A peptide inhibitor derived from p55PIK phosphatidylinositol 3-kinase regulatory subunit: a novel cancer therapy. Mol Cancer Ther. 2008 Dec;7(12):3719–28. doi: 10.1158/1535-7163.MCT-08-0499 19074847

[49] Zhang L, Huang J, Yang N, Greshock J, Liang S, Hasegawa K, et al. Integrative genomic analysis of phosphatidylinositol 3’-kinase family identifies PIK3R3 as a potential therapeutic target in epithelial ovarian cancer. Clin Cancer Res. 2007 Sep 15;13(18 Pt 1):5314–21. doi: 10.1158/1078-0432.CCR-06-2660 17875760

[50] Xia X, Cheng A, Akinmade D, Hamburger AW. The N-terminal 24 amino acids of the p55 gamma regulatory subunit of phosphoinositide 3-kinase binds Rb and induces cell cycle arrest. Mol Cell Biol. 2003 Mar;23(5):1717–25. doi: 10.1128/MCB.23.5.1717-1725.2003 12588990 PMC151709

