# Supplementary figures and images for: ZSTK474 targeting PIK3R3 inhibits the Wilms’ tumor through G0 / G1 phase arrest
Source: PLoS One. 2024 Oct 28;19(10):e0312178. doi: 10.1371/journal.pone.0312178 (PMC11515993; doi:10.1371/journal.pone.0312178)

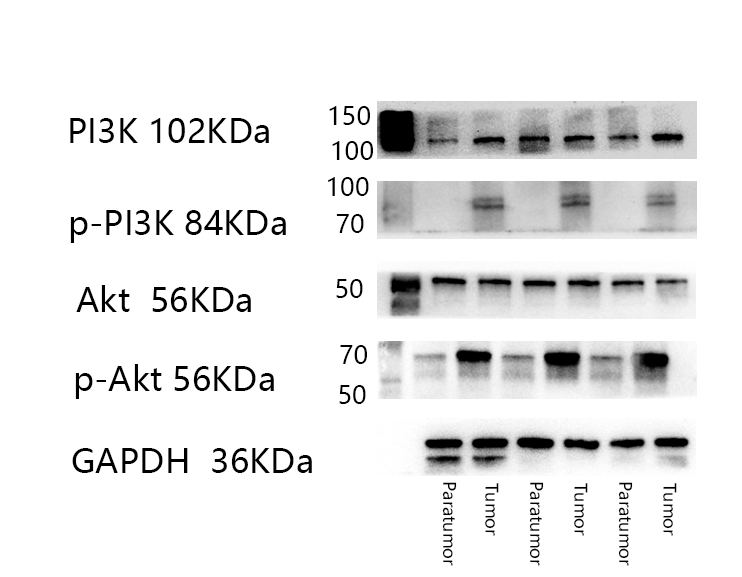

Supplement: S2 File — (ZIP) [file pone.0312178.s002.zip › raw images/raw images 1.tif]

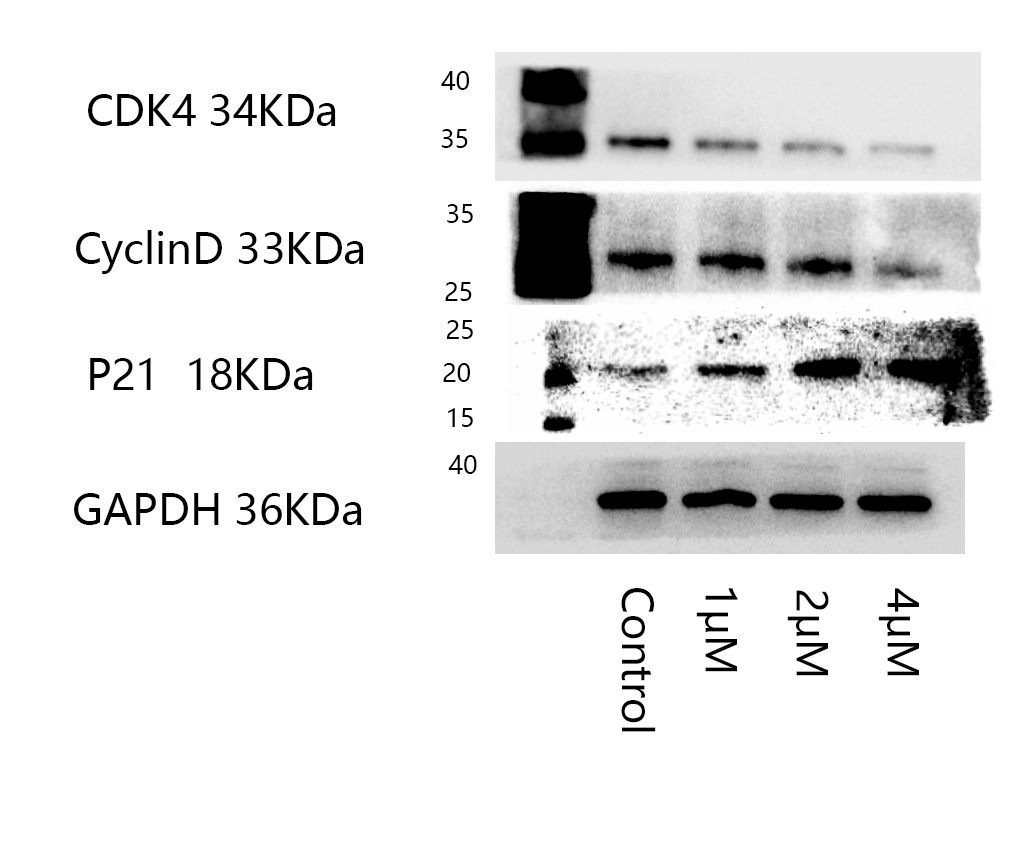

Supplement: S2 File — (ZIP) [file pone.0312178.s002.zip › raw images/raw images 10.tif]

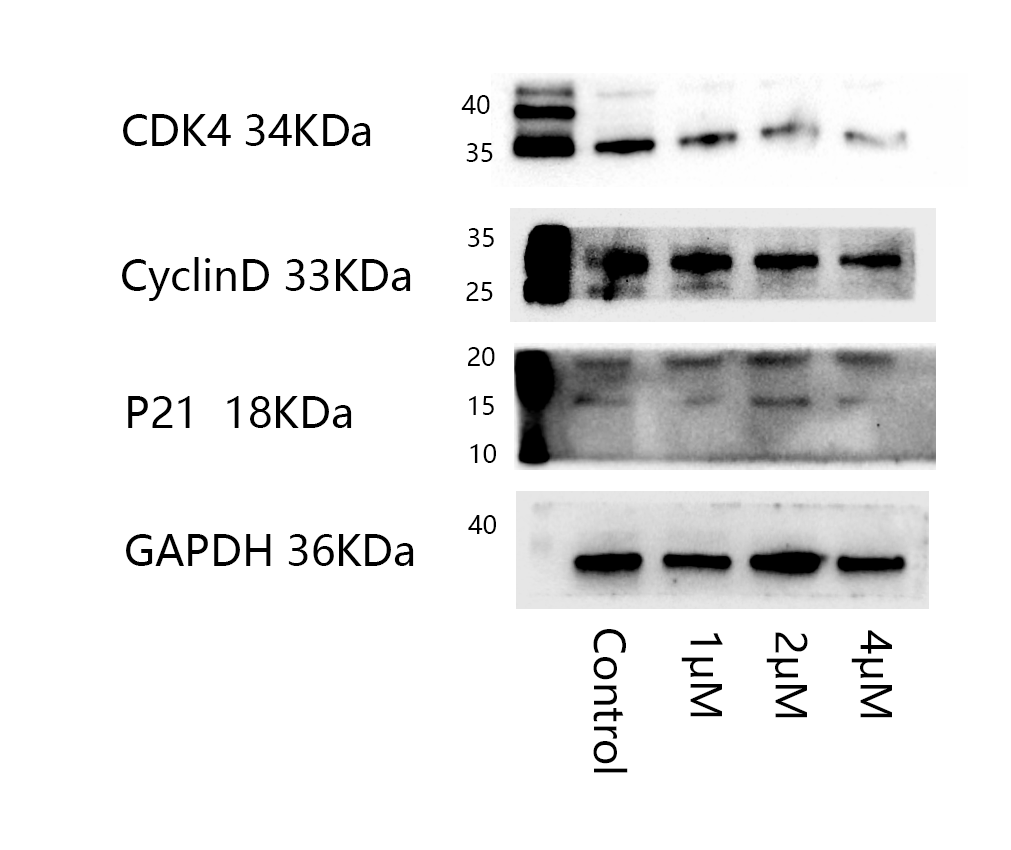

Supplement: S2 File — (ZIP) [file pone.0312178.s002.zip › raw images/raw images 11.tif]

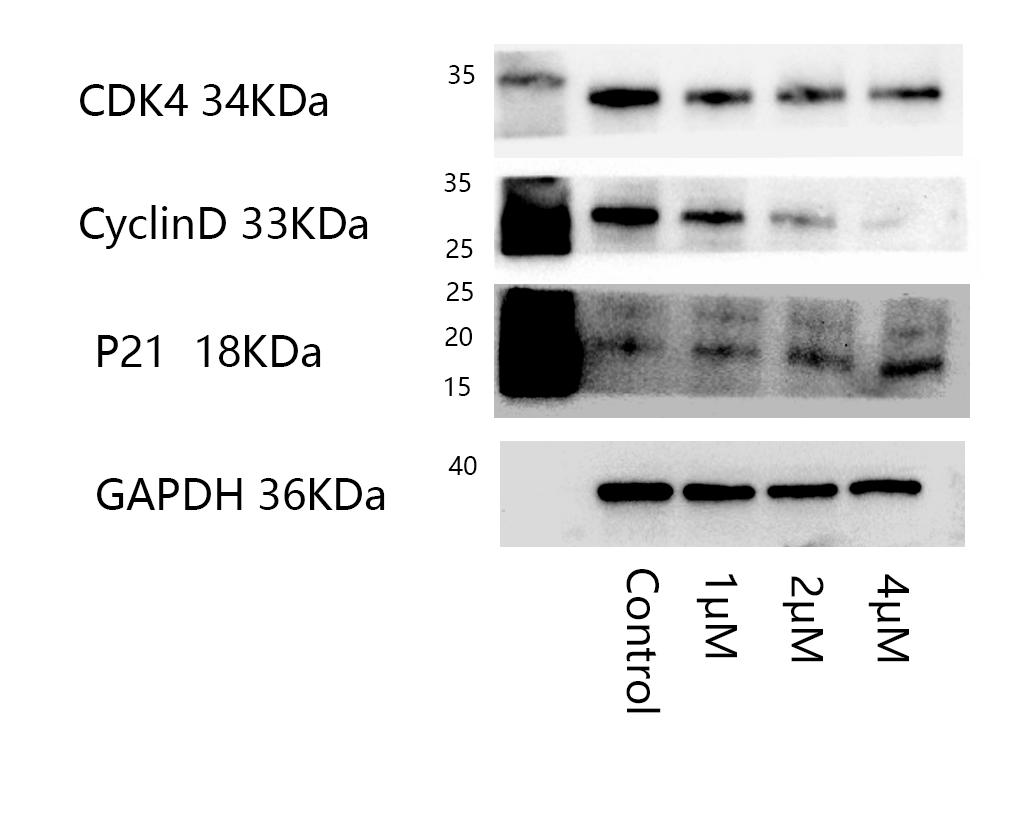

Supplement: S2 File — (ZIP) [file pone.0312178.s002.zip › raw images/raw images 12.tif]

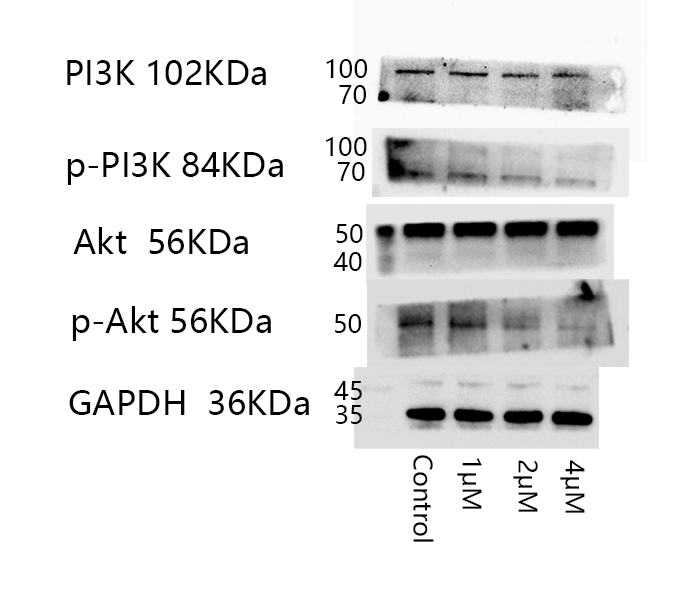

Supplement: S2 File — (ZIP) [file pone.0312178.s002.zip › raw images/raw images 13.tif]

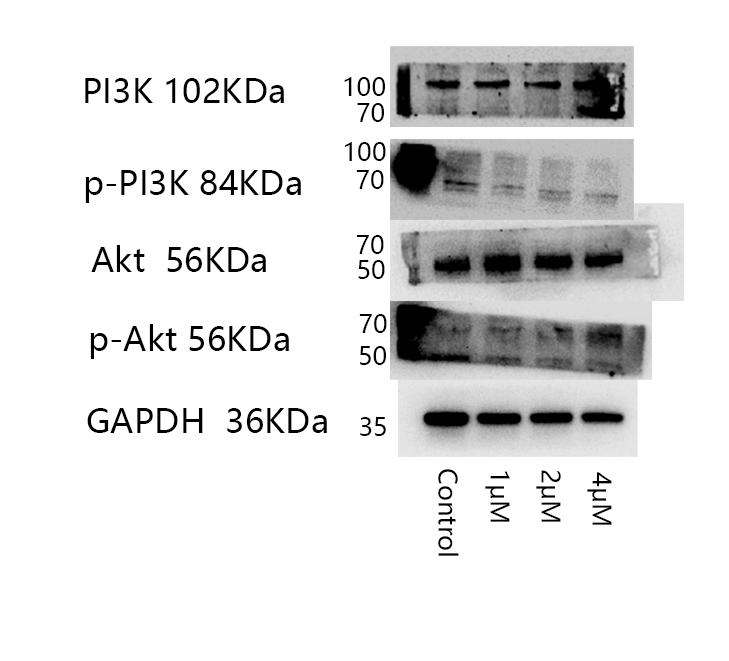

Supplement: S2 File — (ZIP) [file pone.0312178.s002.zip › raw images/raw images 14.tif]

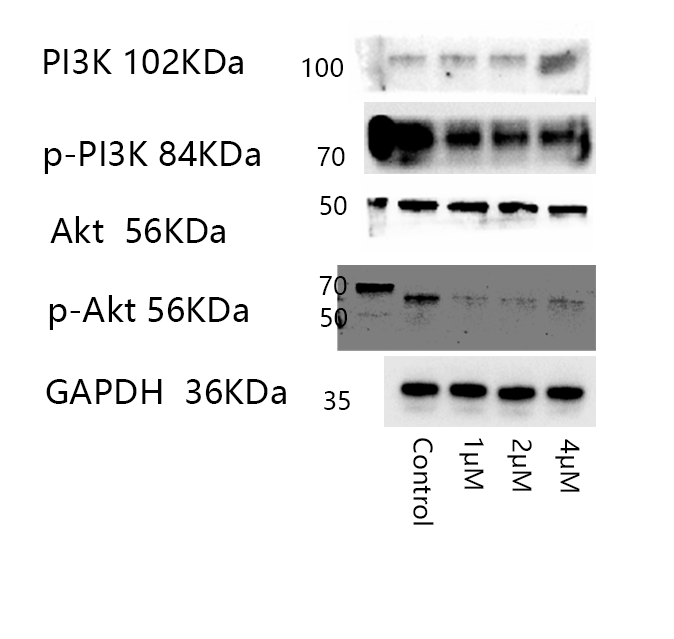

Supplement: S2 File — (ZIP) [file pone.0312178.s002.zip › raw images/raw images 15.tif]

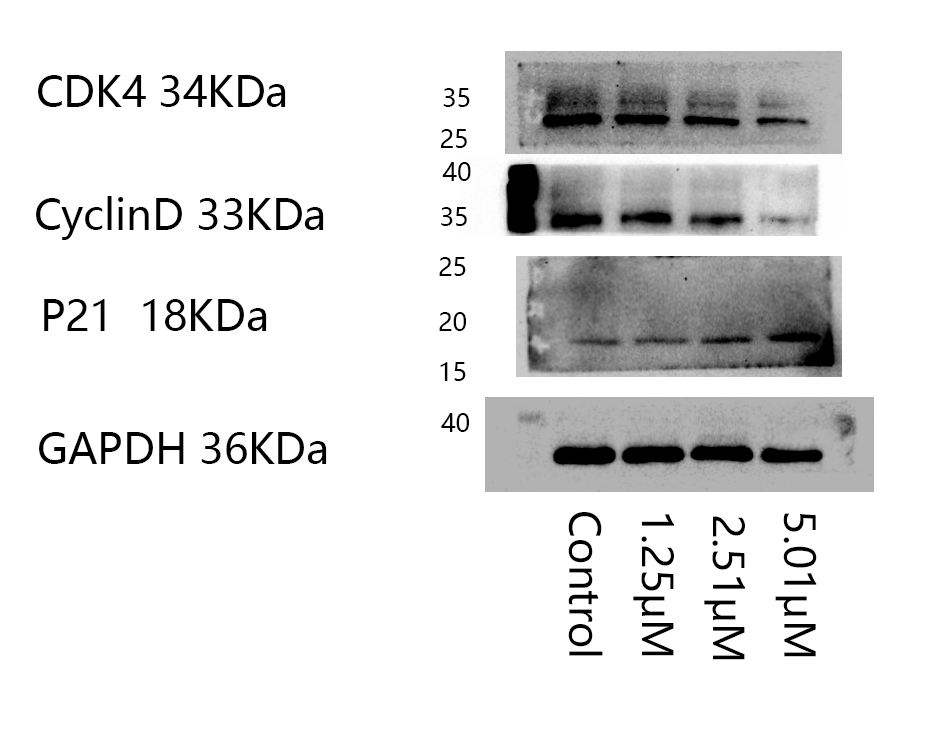

Supplement: S2 File — (ZIP) [file pone.0312178.s002.zip › raw images/raw images 16.tif]

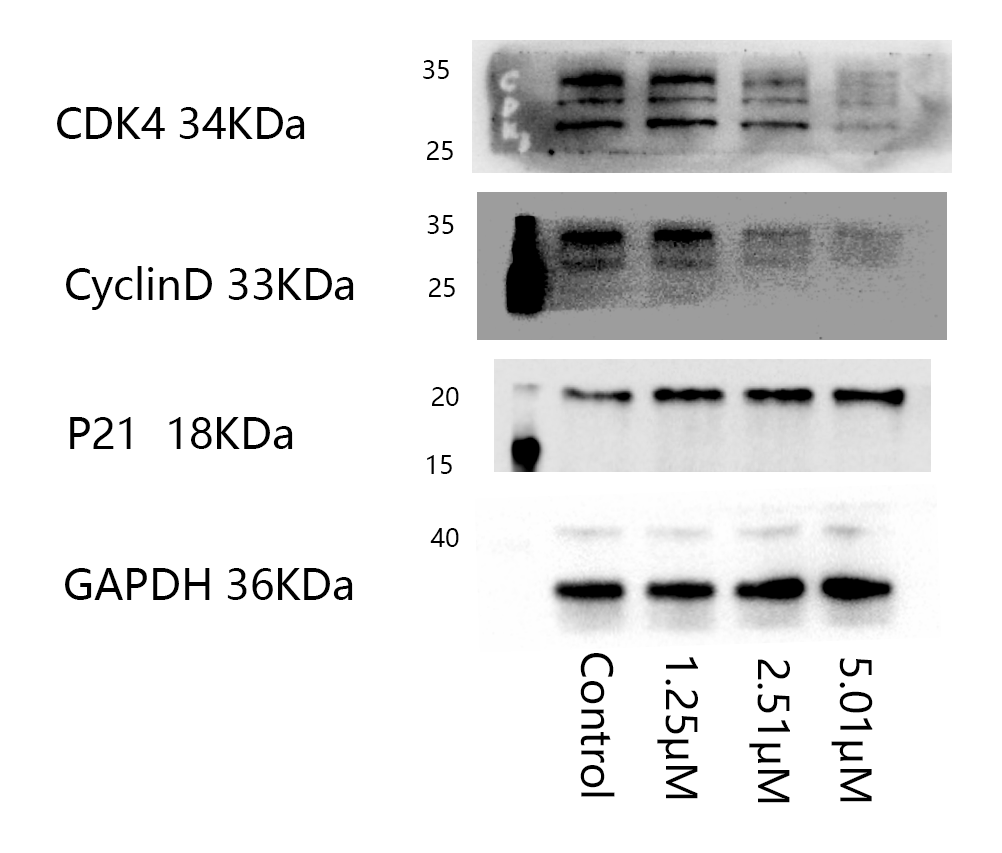

Supplement: S2 File — (ZIP) [file pone.0312178.s002.zip › raw images/raw images 17.tif]

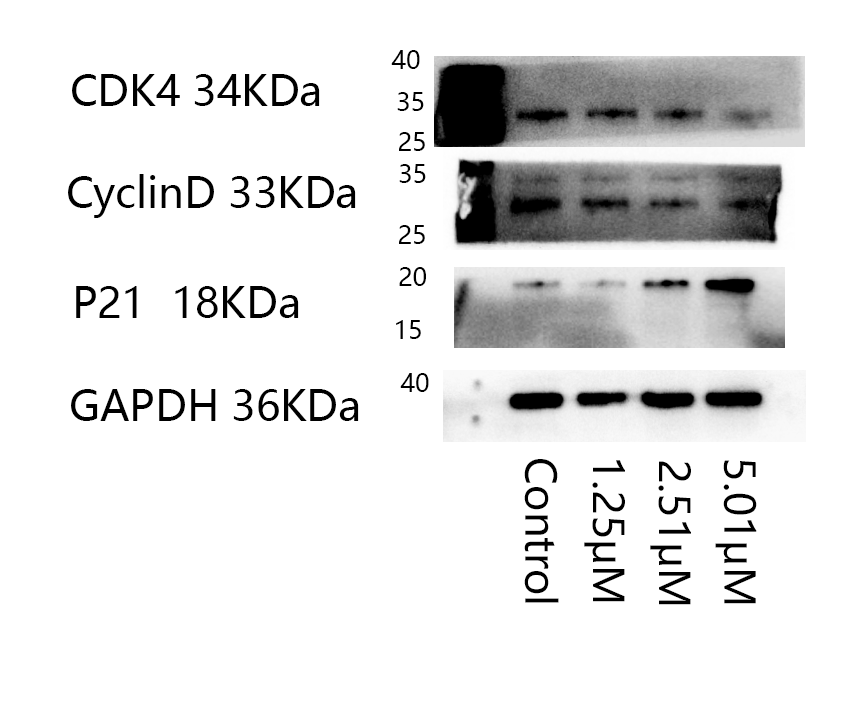

Supplement: S2 File — (ZIP) [file pone.0312178.s002.zip › raw images/raw images 18.tif]

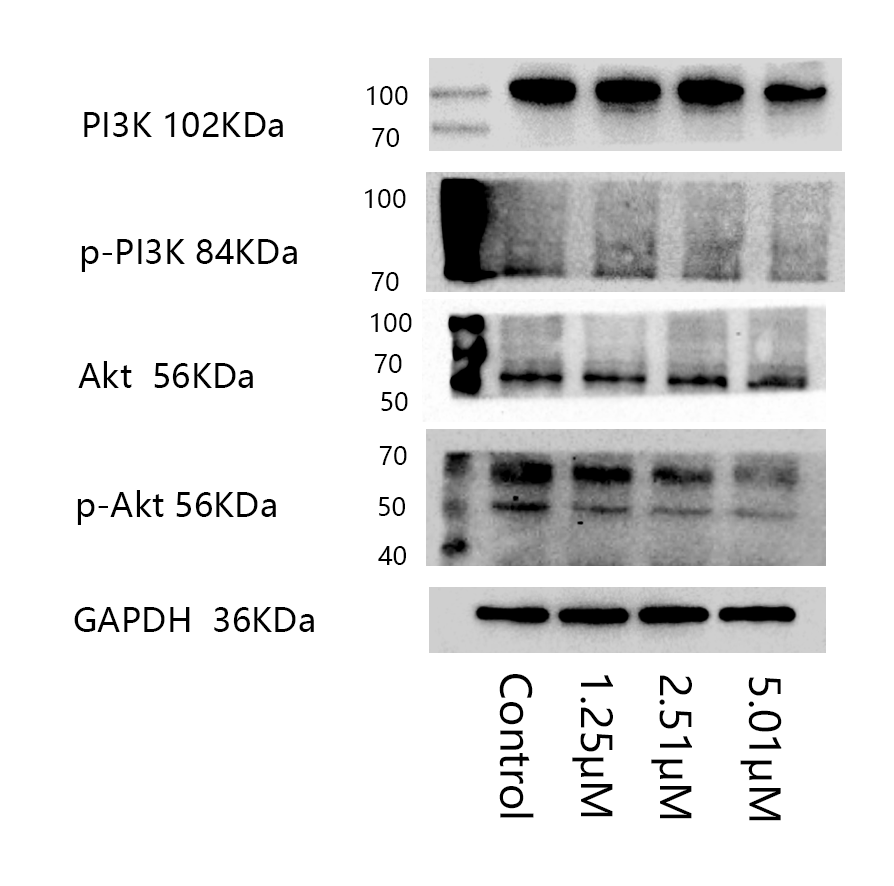

Supplement: S2 File — (ZIP) [file pone.0312178.s002.zip › raw images/raw images 19.tif]

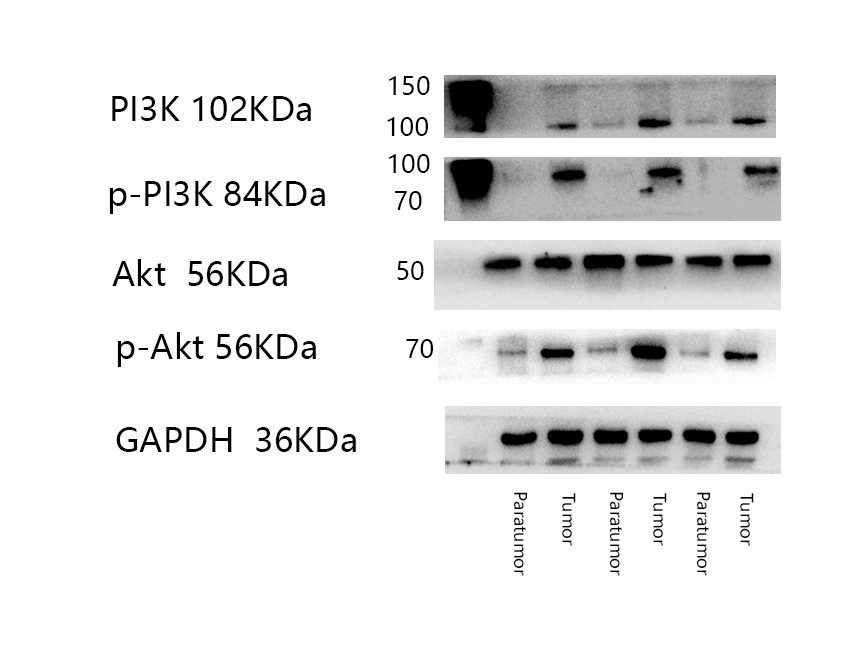

Supplement: S2 File — (ZIP) [file pone.0312178.s002.zip › raw images/raw images 2.tif]

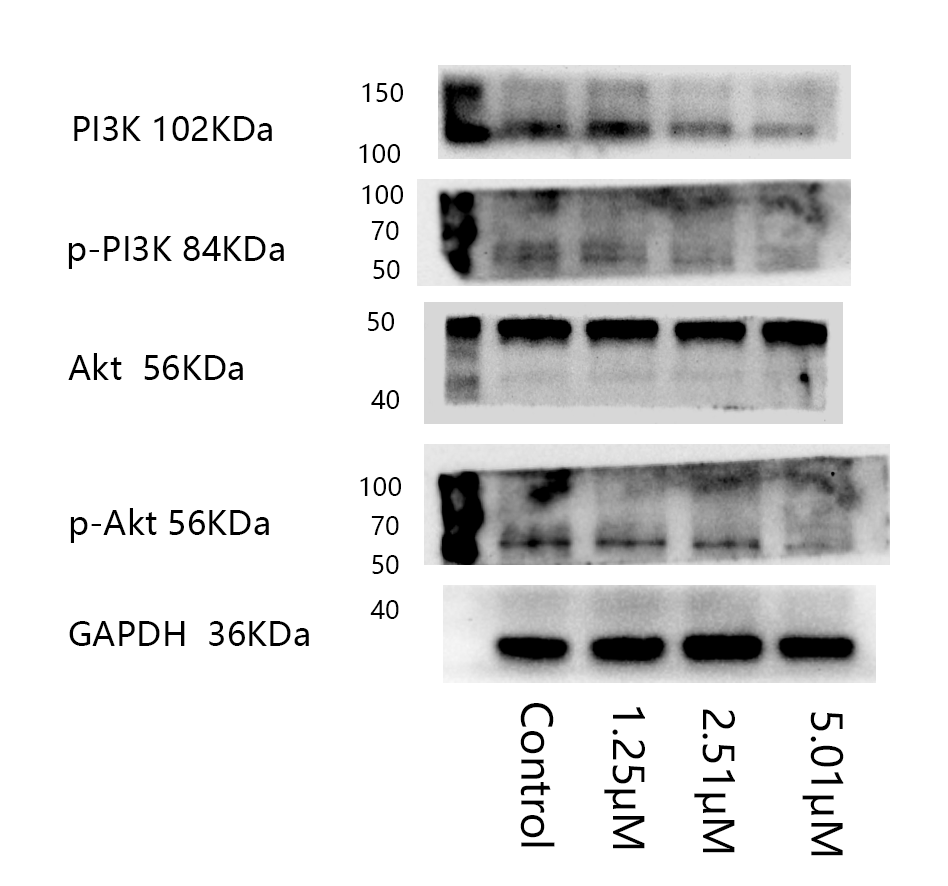

Supplement: S2 File — (ZIP) [file pone.0312178.s002.zip › raw images/raw images 20.tif]

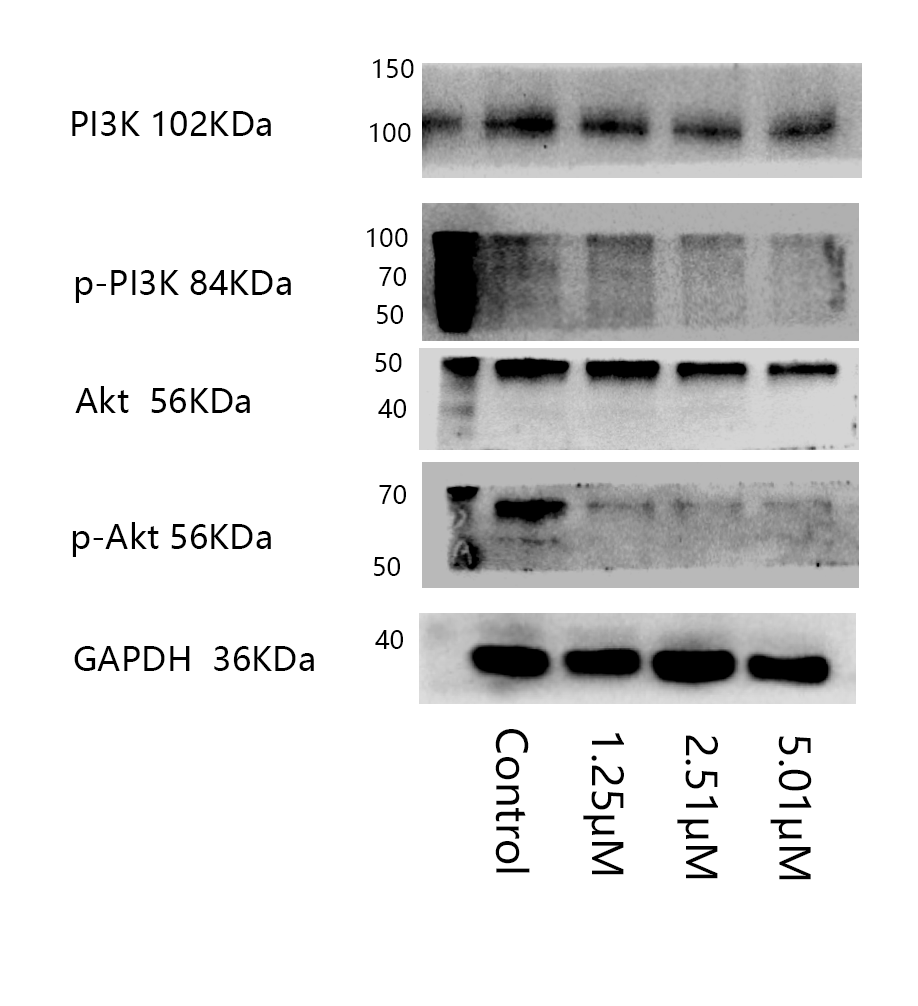

Supplement: S2 File — (ZIP) [file pone.0312178.s002.zip › raw images/raw images 21.tif]

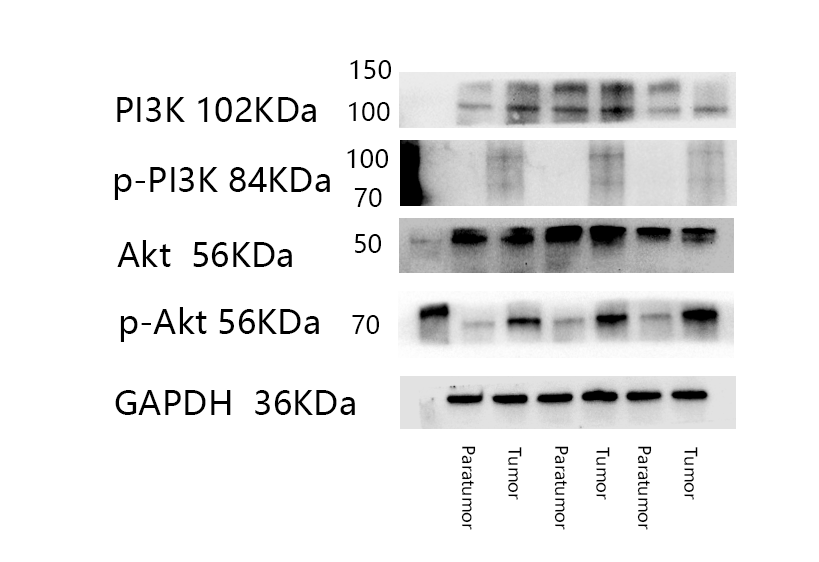

Supplement: S2 File — (ZIP) [file pone.0312178.s002.zip › raw images/raw images 3.tif]

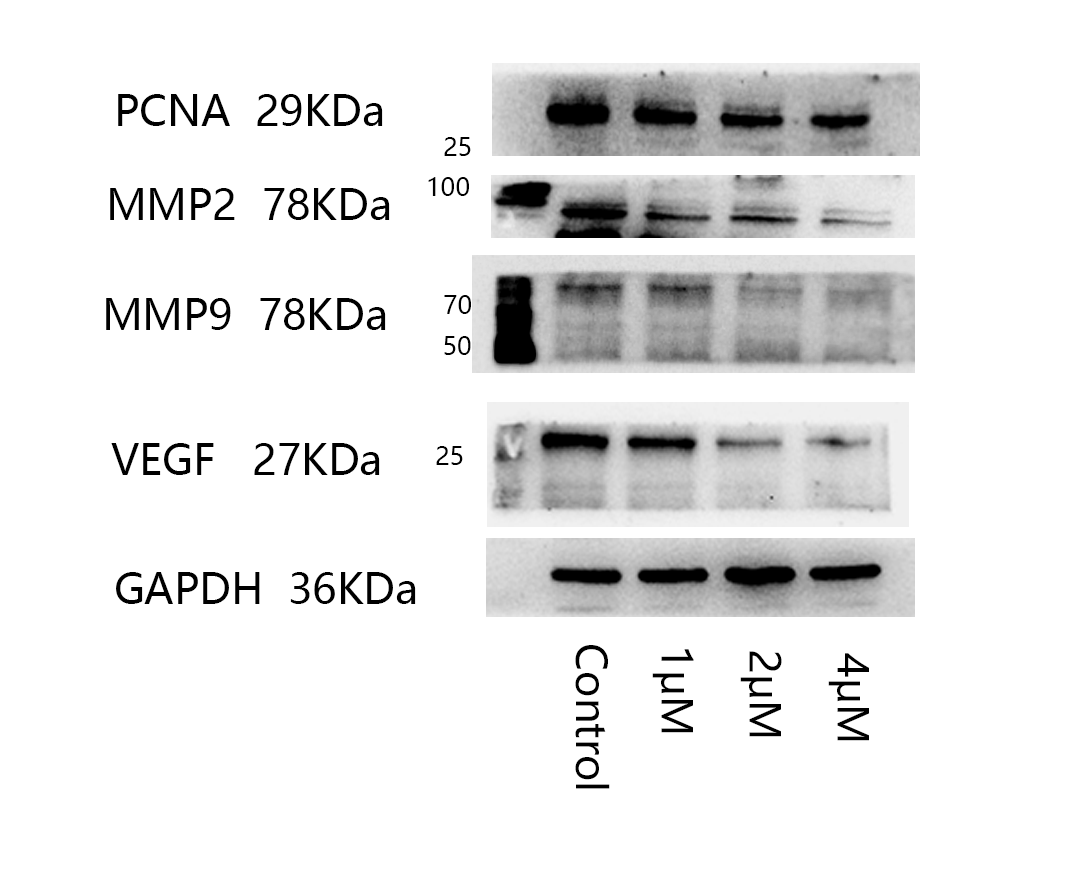

Supplement: S2 File — (ZIP) [file pone.0312178.s002.zip › raw images/raw images 4.tif]

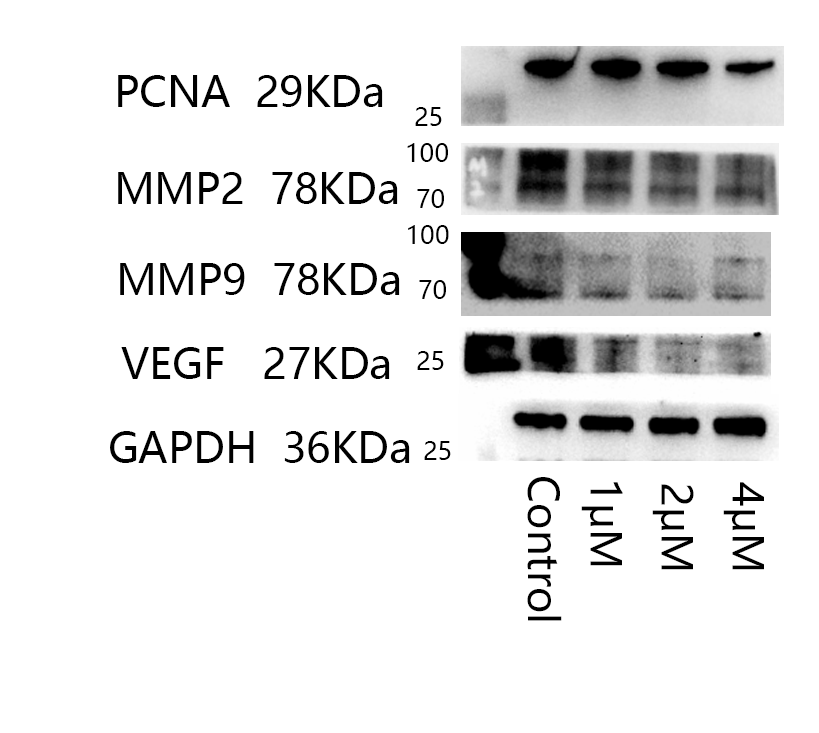

Supplement: S2 File — (ZIP) [file pone.0312178.s002.zip › raw images/raw images 5.tif]

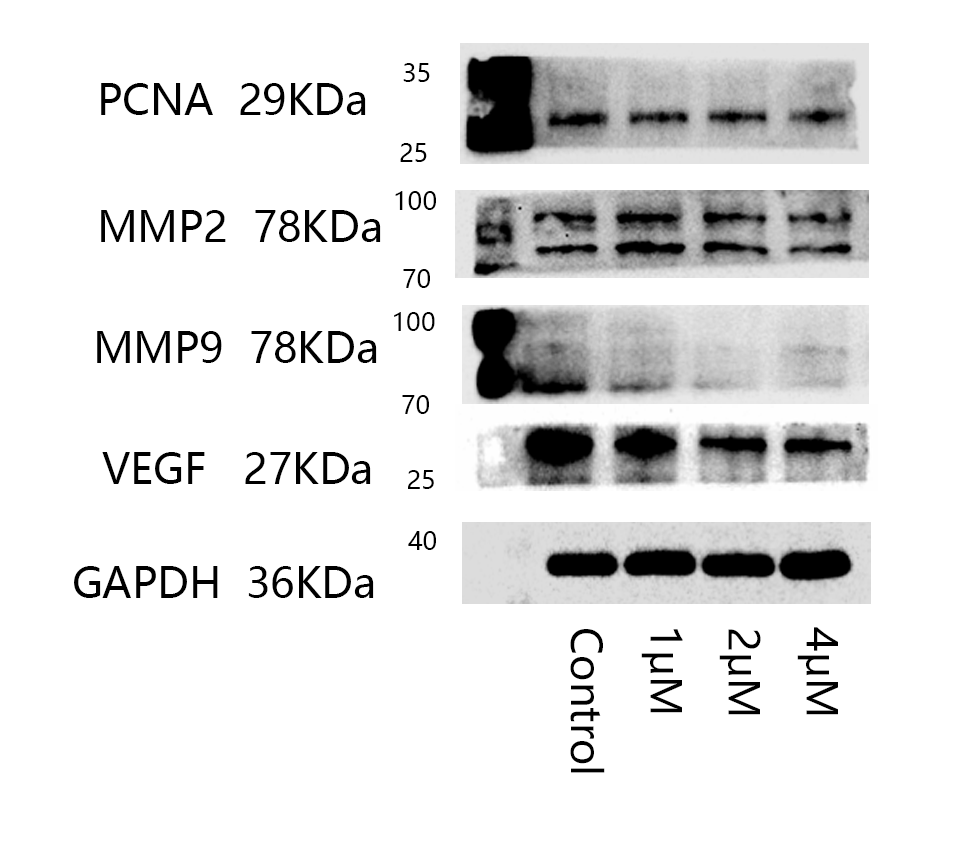

Supplement: S2 File — (ZIP) [file pone.0312178.s002.zip › raw images/raw images 6.tif]

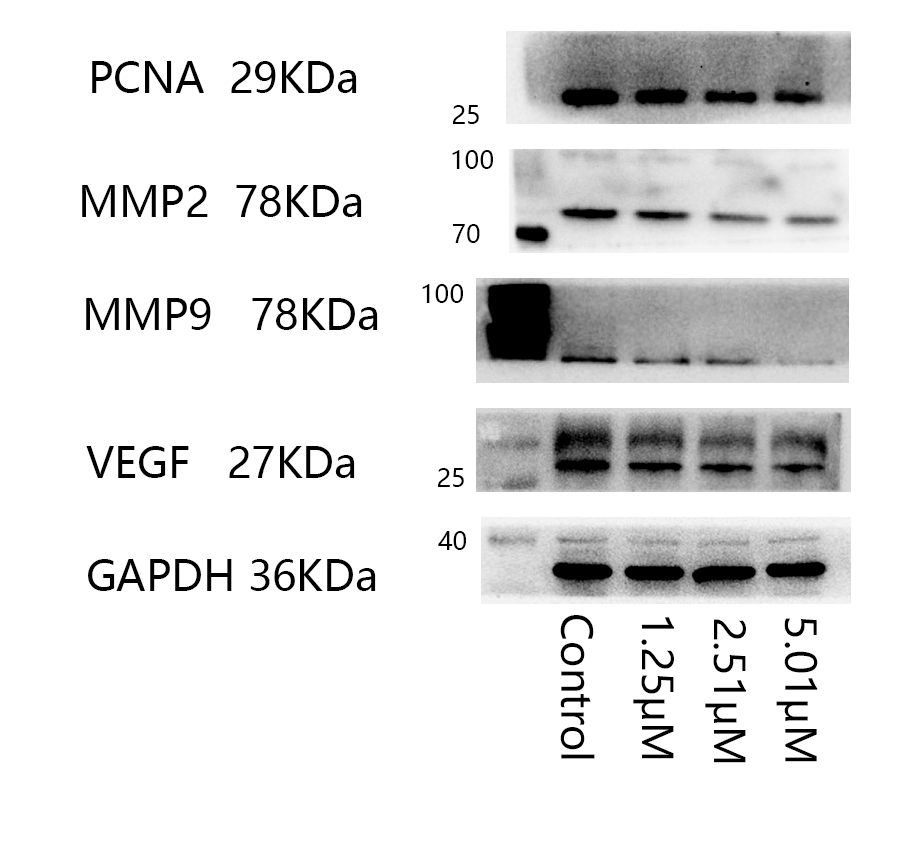

Supplement: S2 File — (ZIP) [file pone.0312178.s002.zip › raw images/raw images 7.tif]

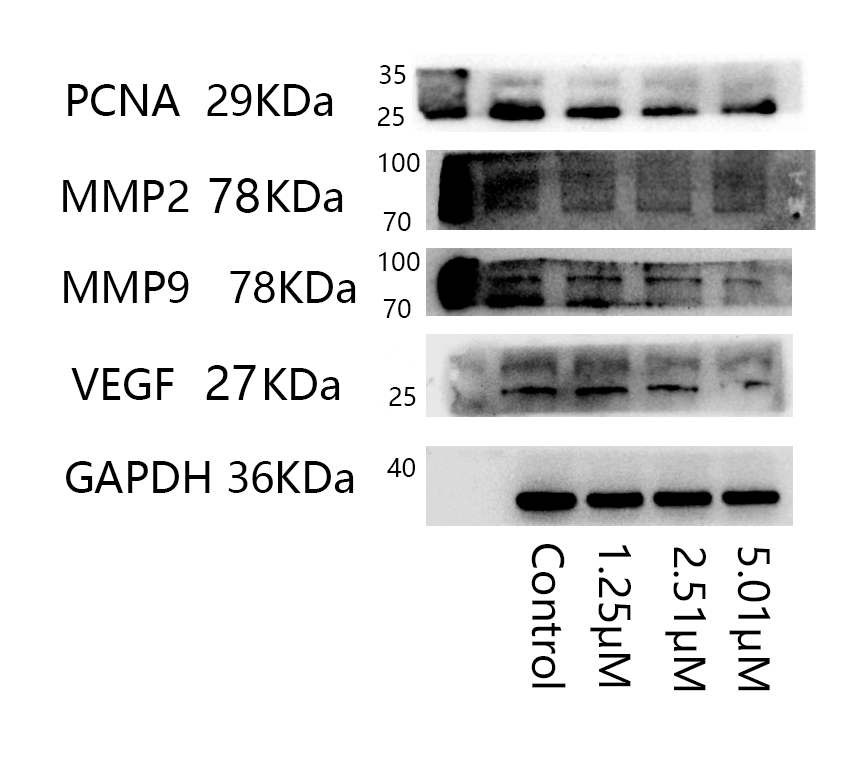

Supplement: S2 File — (ZIP) [file pone.0312178.s002.zip › raw images/raw images 8.tif]

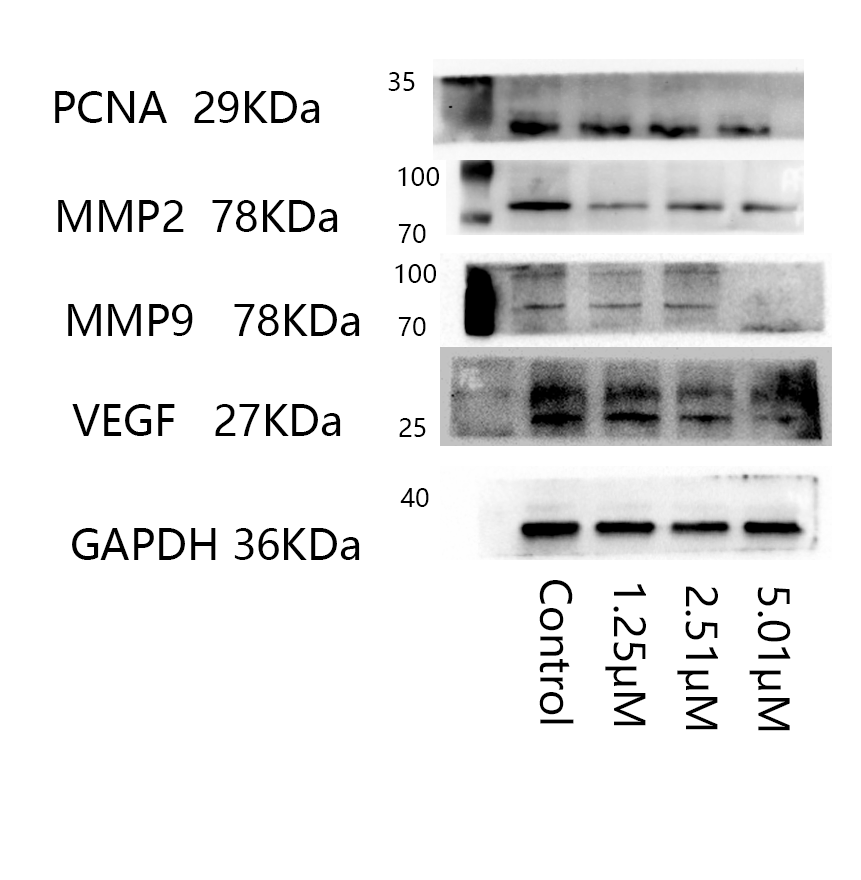

Supplement: S2 File — (ZIP) [file pone.0312178.s002.zip › raw images/raw images 9.tif]
